# 827Spatio-Temporal Quantification of FRET in Living Cells by Fast Time-Domain FLIM: A Comparative Study of Non-Fitting Methods

**DOI:** 10.1371/journal.pone.0069335

**Published:** 2013-07-18

**Authors:** Aymeric Leray, Sergi Padilla-Parra, Julien Roul, Laurent Héliot, Marc Tramier

**Affiliations:** 1 Institut de Recherche Interdisciplinaire, USR 3078 CNRS, Université de Lille-Nord de France, Biophotonique Cellulaire Fonctionnelle, Villeneuve d’Ascq, France; 2 Institut Génétique et Développement de Rennes, UMR 6290 CNRS, Microscopie de Fluorescence Quantitative, Rennes, France; 3 Université de Rennes 1, Université Européenne de Bretagne, SFR Biosit, Faculté de Médecine, Rennes, France; University of California, Irvine, United States of America

## Abstract

Förster Resonance Energy Transfer (FRET) measured with Fluorescence Lifetime Imaging Microscopy (FLIM) is a powerful technique to investigate spatio-temporal regulation of protein-protein interactions in living cells. When using standard fitting methods to analyze time domain FLIM, the correct estimation of the FRET parameters requires a high number of photons and therefore long acquisition times which are incompatible with the observation of dynamic protein-protein interactions. Recently, non-fitting strategies have been developed for the analysis of FLIM images: the polar plot or “phasor” and the minimal fraction of interacting donor *mf_D_*. We propose here a novel non-fitting strategy based on the calculation of moments. We then compare the performance of these three methods when shortening the acquisition time: either by reducing the number of counted photons *N* or the number of temporal channels *N_ch_*, which is particularly adapted for the original fast-FLIM prototype presented in this work that employs the time gated approach. Based on theoretical calculations, Monte Carlo simulations and experimental data, we determine the domain of validity of each method. We thus demonstrate that the polar approach remains accurate for a large range of conditions (low *N*, *N_ch_* or small fractions of interacting donor *f_D_*). The validity domain of the moments method is more restricted (not applicable when *f_D_*<0.25 or when *N_ch_* = 4) but it is more precise than the polar approach. We also demonstrate that the *mf_D_* is robust in all conditions and it is the most precise strategy; although it does not strictly provide the fraction of interacting donor. We show using the fast-FLIM prototype (with an acquisition rate up to 1 Hz) that these non-fitting strategies are very powerful for on-line analysis on a standard computer and thus for quantifying automatically the spatio-temporal activation of Rac-GTPase in living cells by FRET.

## Introduction

Förster Resonance Energy Transfer (FRET) is a photo-physical phenomenon in which energy is non-radiatively transferred from one excited fluorescent donor molecule to a nearby acceptor. It depends on both the distance and the relative orientation of the two fluorophores (donor and acceptor) and occurs efficiently when the distance between the two molecules is less than approximately 10 nm [1], a distance comparable to the dimensions of biological macromolecules. Measuring this phenomenon has then been largely used for detecting protein–protein interactions and protein conformational changes inside living cells (for review see [2], [3], [4]).

Since FRET affects the photo-physical properties (fluorescence intensity and lifetime) of both donor and acceptor, it can be measured with different techniques [5], [6], [7], [8]. In this work, we investigate FRET by measuring the donor fluorescence lifetime with fluorescence lifetime imaging microscopy (FLIM). This technique is advantageous for measuring FRET compared to the intensity based approaches since the lifetime is a spectroscopic property which is insensitive to the donor concentration, the donor-acceptor stoichiometry and the excitation intensity fluctuations.

Lifetime imaging has been successfully performed with both frequency domain (FD) [9], [10] and time domain (TD) methods [11], [12]. In the former case, the sample is excited using a sinusoidally modulated source and the fluorescence lifetime is calculated from the phase shift or the modulation depth of the fluorescence signal relative to the excitation light.

In this article, the fluorescence lifetime is measured with the TD method. In this case, the sample is excited using a series of short laser pulses and the resulting intensity decay histograms *I(t)* are acquired. From these experimental data, the fluorescence lifetime is often estimated by minimizing the errors between the recorded decay profiles and a mathematical model (fitting methods). In FRET experiments, the probability density function is modeled by a sum of two exponentials, since the fluorescence signal emitted by the sample is a mixture of the signal originating from the donor alone and from the donor in the presence of the acceptor:
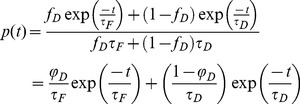
(1)where φ*_D_* is the fractional contribution of the donor in interaction defined by
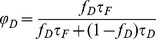
(2)where f_D_ is the proportion of donor in interaction, τ_D_ is the donor lifetime and τF is the lifetime of the donor when FRET occurs.

The standard fitting method has been largely used for estimating all parameters (*f_D_, τ_D_* and *τ_F_*) in FRET experiments [1]. However their correct determination with this method is time consuming and requires a high number of photons (more than 100000 photons according to Köllner & Wolfrum [13]) which implies a long acquisition time that is not compatible with the observation of dynamic molecular events in living cells [14].

In order to speed up and simplify the analysis of FLIM images, alternative methods based on non-fitting approaches [15] have been developed recently: the polar plot or phasor [16], [17] and the minimal fraction of interacting donor [14].

The polar representation was initially described by Jameson et al. [18] and then successively improved by different groups [19], [20], [21]. In the TD, the polar approach consists in calculating the Fourier sine and cosine transforms of all experimental intensity histograms (called polar coordinates) in order to convert the FLIM image into a scatter diagram whose position gives a fast and visual indication on the fluorescence lifetime and greatly facilitates the analysis of FLIM data [16]. Recently, we have demonstrated that it is also possible to retrieve quantitatively the FRET parameters from analytical expressions incorporating the polar coordinates with a fully non-fitting approach [22].

The minimal fraction of interacting donor (*mf_D_*) introduced by us [14] is an alternative non-fitting method that allows analytical determination of the minimal relative concentration of interacting proteins from the mathematical minimization of *f_D_*.

In this work, we describe an additional non-fitting approach called the moments method based on the calculation of the first and second order moments of the probability density function p(t) which gives access to the FRET parameters (*f_D_* and *τ_F_*). A variant of this method has already been proposed by Isenberg and Dyson for resolving single fluorescence decay [23]. However, it requires an iterative fitting procedure for corrections estimation and to the best of our knowledge it was never applied to analyze FLIM image. The moments method that we introduce in this work is a fully non-fitting approach that can be automated.

The major advantage of these three methods resides in the fact that the FRET parameters are deduced from simple mathematical operations that can be performed on-line on a standard computer and thus be fully automated. These strategies should thus be particularly well adapted for fast-FLIM FRET experiments but to the best of our knowledge this issue has never been exhaustively investigated in the literature.

In this article, we evaluate the performance of these non-fitting approaches when utilizing fast FRET FLIM experiments. According to the experimental set up used, the acquisition time in TD FLIM experiments may be shortened either by reducing the number of photons or the number of temporal channels. When the number of counted photons is low, we demonstrate computationally that the FRET parameters estimated with the standard fitting method are directly dependent on the initial conditions, prohibiting this approach for automated quantification of FRET FLIM experiments. We have also investigated the performance of these non-fitting strategies when either the number of photons or the number of temporal channels is low and we have determined the domain of validity of each method from both Monte Carlo simulations and experimental data acquired on model solutions exhibiting two lifetimes at different ratios with our fast-FLIM prototype based on time-gated images. We finally applied these non-fitting strategies to quantify Rac-GTPase activity by using a PBD assay [24] and we have successfully evaluated the spatio-temporal activation of Rac (a small GTPase which regulates the formation of ruffles and filopodia in polarized cells) by measuring the FRET signal with our fast-FLIM prototype capable of acquiring FLIM images at high speed (∼1 FLIM image/sec).

## Materials and Methods

### Non-fitting approaches

- *mf_D_*.

**Figure 1:**
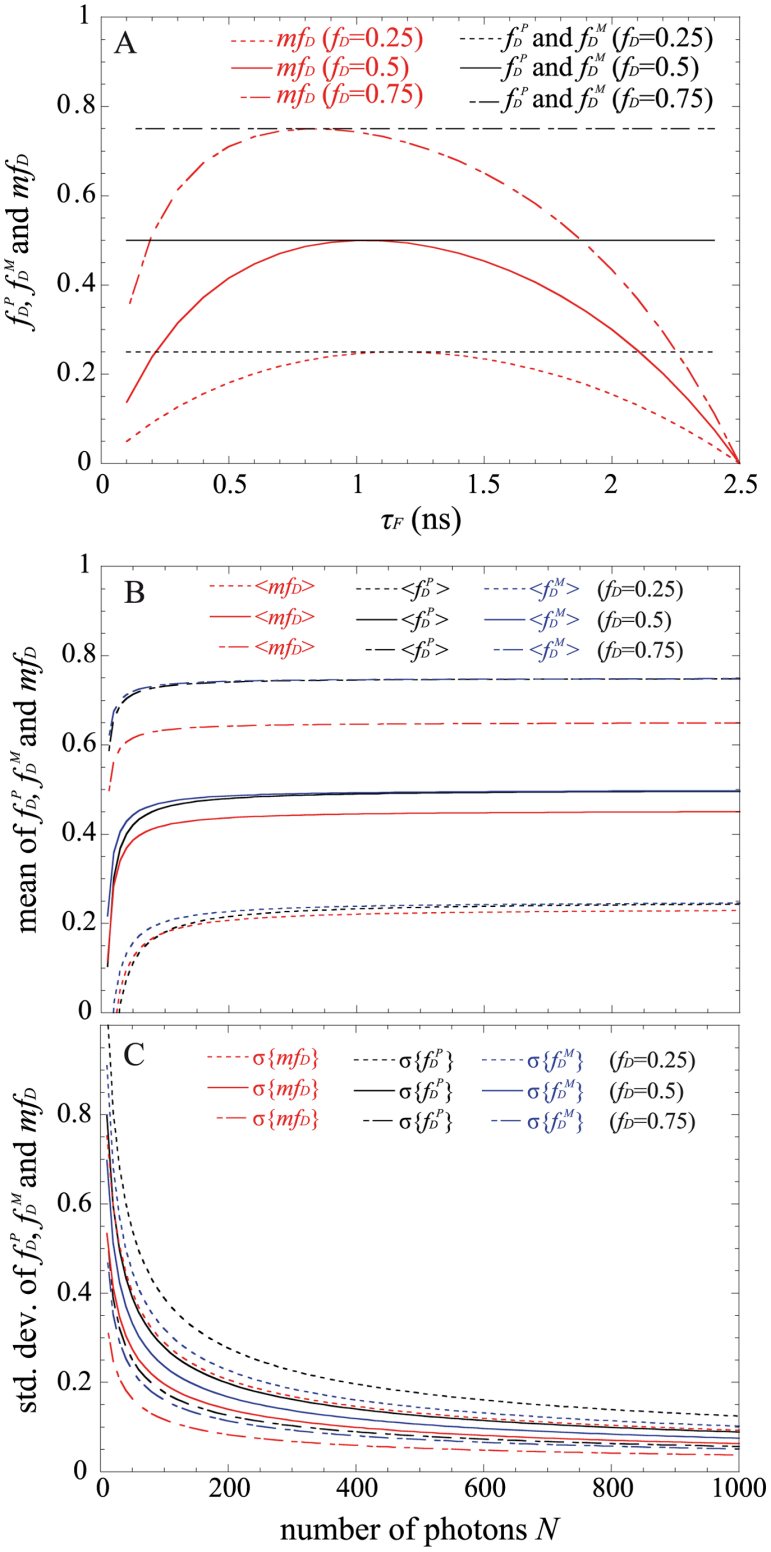
Theoretical fractions of interacting donor calculated with the polar approach *f_D_^P^* or the moments method *f_D_^M^* (in black) and theoretical *mf_D_* values (in red) as a function of the lifetime of the donor in presence of the acceptor *τ_F_* (for a donor lifetime of 2.5 ns). Three distinct *f_D_* values were considered in (A). We have also plotted the means in (B) and the standard deviations in (C) of *f_D_^P^* in black (deduced from Eqs. 15 and 16), those of *f_D_^M^* in blue (deduced from Eqs. 18 and 19) and those of *mf_D_* in red (deduced from Eqs. 13 and 14) as a function of the total number of photons *N*. The following FRET parameters were used: *τ_F_* = 1.5 ns, *τ_D_* = 2.5n s and *f_D_* = 0.25, 0.5 and 0.75.

The minimal fraction of interacting donor *mf_D_* was introduced by us in 2008 [14]. Briefly, if the donor intensity decay is mono-exponential (i.e. eGFP or mTFP1 [25]); a two populations system (FRET and no-FRET species) with a narrow distribution of FRET efficiencies can be assumed when FRET occurs. In this case a bi-exponential intensity decay can be employed to describe the fluorescence decay (cf. Eq. 1) and the minimal fraction of interacting donor is given by
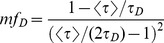
(3)where <*τ>* is the mean lifetime defined by



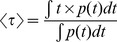
(4)This parameter has already been exhaustively compared with the true fraction of interacting donor (*f_D_*) in [14]. Briefly, as indicated in Fig. 1A, *mf_D_* is equal to *f_D_* only for one particular FRET lifetime *τ_F_* (indeed when *τ_F_* = <*τ*>/2) but the error between *mf_D_* and *f_D_* remains confined for a relatively large range of *τ_F_* (around 500 ps).

- Polar approach.

The theory of the polar approach for TD FLIM experiments has been detailed previously [16], [26]. Briefly, each acquired intensity histogram in TD FLIM experiments is converted into [*u*;*v*] coordinates. These *u* and *v* coordinates are respectively the cosine and sine transforms of the fluorescence intensity decay *p(t)* which are defined by
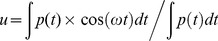
(5)

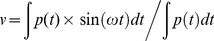
(6)where *ω* is the laser repetition angular frequency.

Recently, we have demonstrated that it is possible to retrieve quantitatively the FRET parameters from these polar coordinates [22]. In fact, for a bi-exponential intensity decay (and in the case of a single exponential donor), the fraction of interacting donor *f_D_* and the fluorescence lifetime of the donor in presence of the acceptor *τ_F_* can be analytically expressed as
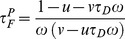
(7)

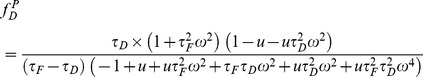
(8)


We have represented this analytical fraction of interacting donor as a function of *τ_F_* in Fig. 1A and we can clearly notice that the analytical *f_D_^P^* corresponds exactly to the true *f_D_* for all lifetime values *τ_F_* comprised between 0 and *τ_D_*.

- Moments method.

We propose an alternative method for estimating the fraction of interacting donor *f_D_* and the fluorescence lifetime of the donor in presence of the acceptor *τ_F_* for a bi-exponential intensity decay. Our method is based on the calculation of the moments of the first and second order which are defined by
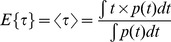
(9)

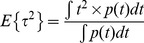
(10)


By simply resolving the system of equations 9 and 10, a straightforward calculation leads to
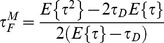
(11)

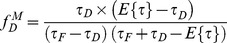
(12)


The analytical fraction of interacting donor *f_D_^M^* is represented as a function of *τ_F_* in Fig. 1A; it is equal to the true fraction of interacting donor when the lifetime *τ_F_* is comprised between 0 and *τ_D_*.

### Monte Carlo Simulations

For generating TD FLIM images with controlled parameters, we have performed Monte Carlo simulations on a standard computer. More details on the algorithm can be found in [27], [28]. In order to be as close as possible of the experimental conditions, we consider a laser repetition frequency of 80 MHz which corresponds to a total window width of 12.5 ns. All the simulated inte ity decays consist of two components whose lifetimes are respectively *τ_F_* = 1.5ns and *τ_D_* = 2.5 ns and three distinct fractions of interacting donor were used *f_D_* = 0.25; 0.5 and 0.75. For each condition, we have simulated a FLIM image of 64×64 pixels corresponding to 4096 intensity decays and each simulated decay is composed of *N* photons divided into *N_ch_* temporal channels.In this work, we have simulated lifetime images acquired with two largely used TD FLIM systems: the time correlated single photon counting (TCSPC) and the time gated system. For TCSPC simulations, the measurement window which is divided into 64 temporal channels is limited to 12.5 ns and the offset is neglected because it is generally less than few photons per pixel. The full width half maximum (FWHM) of the simulated Gaussian instrumental response function (IRF) with the TCSPC technique is fixed to 32 ps, as measured by Waharte et al. [12]. For time gated simulations, we consider that the measurement window of 12.5 ns is divided into *N_ch_* contiguous gates with variable width *w* = 12.5/*N_ch_*. The FWHM of the simulated IRF is fixed to 200 ps corresponding to the measured rising time of our time gated system. To simulate the intensifier noise, we added to each pixel a Poisson distributed offset of 190 photons (corresponding to 1600 grey levels in our time gated system). The simulated FLIM images are finally smoothed with a 3×3 average filter in order to obtain a comparable signal to noise ratio than those of our time gated system. For investigating the performance of all FLIM image analysis strategies during fast-FLIM FRET experiments, we have considered several total numbers of photons *N* and several numbers of gates *N_ch_*.

### Experimental Setup

**Figure 2:**
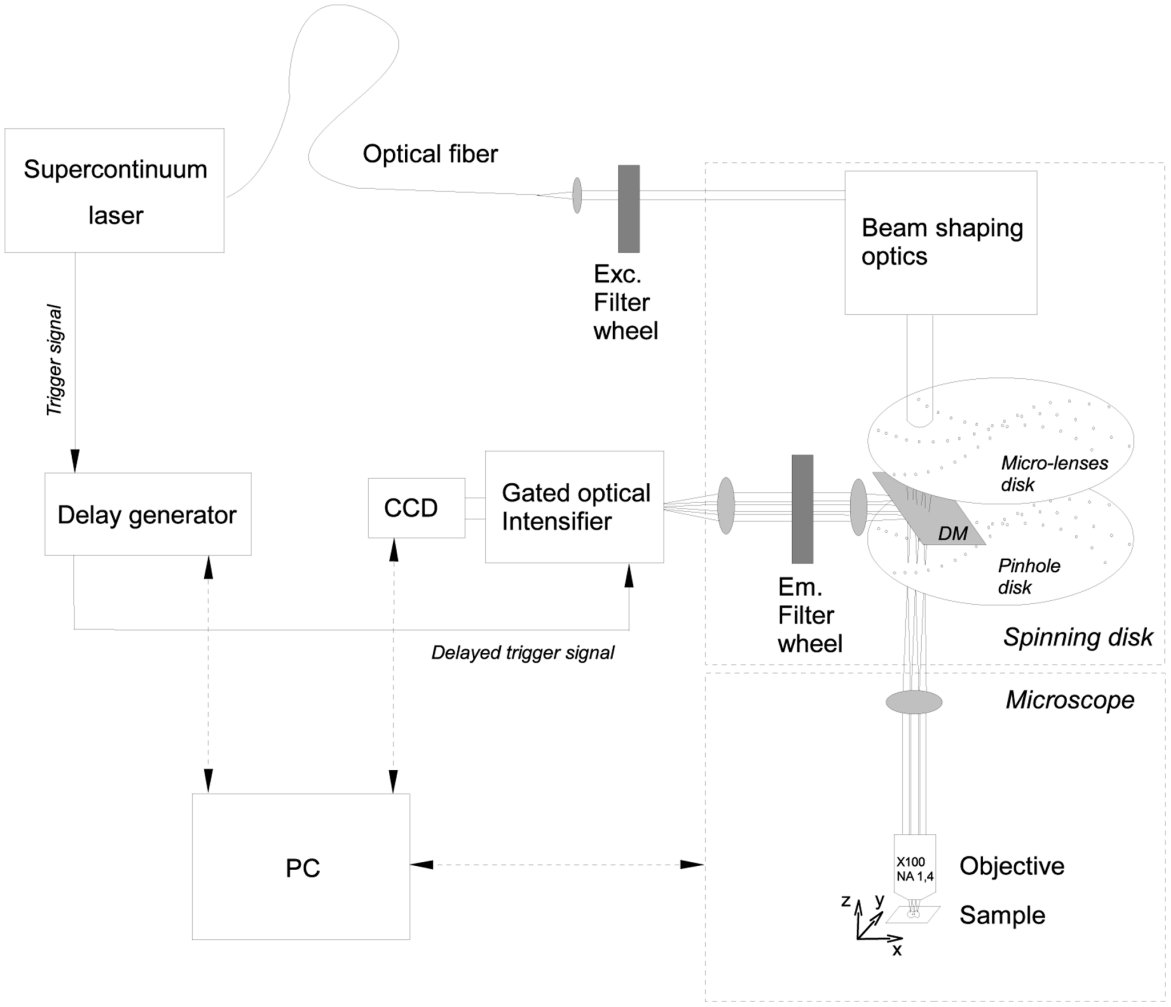
Fast-FLIM scheme. The supercontinuum laser is collimated out of the fiber, spectrally filtered (473–491 nm) and injected into the Yokogawa spinning disk system where shaping optics extend the beam. The micro-lenses disk creates a multitude of beams focused in the pinholes of the coupled disk conjugated with the sample plane. The emitted fluorescence is selected by a dichroic mirror DM (transmission peak at 488 nm) and an emission filter (500–550 nm), and converted into electrons with the photocathode of the intensifier. Each laser pulse triggers the photocathode so that it runs as an ultra-fast shutter (time gate of 2.25 ns at 80 MHz). The electrons are amplified by a micro channel plate and converted back into light with a phosphorescent screen. The photons are finally acquired with a CCD camera (with binning 3×3) and the fluorescent images are saved. A home-made MetaMorph user program called Flimager (MFQ, IGDR) was developed for both controlling the complete system (delay generator, CCD camera and microscope), and calculating the FLIM images on-line.

Our fast-FLIM system combines a supercontinuum laser, a spinning disk system to improve spatial resolution and provide optical sectioning and a fast-gated intensifier coupled to a CCD camera for rapid FLIM acquisition (Fig. 2). The supercontinuum laser (Fianium SC400-6) provides a wide spectrum from 400 to 2400 nm with a high power in the visible range (3 mW/nm) which is well adapted for FLIM measurements when it is combined with a multifocal system. The multifocal illumination of the sample is performed by a spinning disk system (Yokogawa CSU-X1) implemented on an inverted microscope (Leica DMI6000). The excitation light is spectrally filtered before illuminating the sample through an oil immersion objective (100×, NA 1.4, Leica). The fluorescence decay is acquired with a CCD camera (CoolSnap hq^2^, Photometrics) coupled to a fast gated intensifier (PicoStar, LaVision, Kentech Instruments) triggered with an electronic signal coming from the laser. This electronic signal is sequentially delayed by a programmable delay generator for obtaining a stack of time-correlated images. Each image which corresponds to a temporal width of 2.25 ns is acquired with an exposure time that usually varies between 20 and 100 ms. Our system is fully controlled by MetaMorph (Molecular Devices) and the FLIM acquisitions are driven by a homemade MetaMorph program (Flimager) that calculates the mean lifetime (cf. Eq. 4) on-line from a background-subtracted and smoothened (with a 3×3 average filter) fluorescence stack of images and provides a FLIM acquisition rate up to 1 image/s allowing to follow the spatio-temporal evolution of the protein-protein interactions occurring in the sample.

### FLIM Image Analysis

For analyzing the TD-FLIM images with the standard fitting method, we have used the well known least square method (with Levenberg-Marquardt algorithm) for minimizing the difference between the experimental data and the theoretical model [29]. All temporal histograms were fitted with a two components exponential model and the first lifetime *τ_D_* was fixed to the donor lifetime (which is equal to 2.5 ns for the simulated FLIM images). The following parameter constraints (min lifetime: 0 ns; max lifetime: 2.5 ns; max ratio: 1) and standard algorithmic settings (10 iterations, Δχ^2^ = 0.001) were used.

The TD FLIM images have also been analyzed with a custom-made software named MAPI (IRI, USR 3078 CNRS, BCF, available on request: http://biophotonique.univ-lille1.fr/spip.php?rubrique60) for investigating the performance of the non-fitting methods (*mf_D_*, polar approach and moments method). This software allows computing the Fourier sine and cosine transforms of all temporal histograms and calculating the proportion of interacting donor and the donor lifetime in presence of the acceptor deduced from both the polar approach (cf. Eqs. 7 and 8) and the moments method (cf. Eqs. 11 and 12) when the donor lifetime is fixed. MAPI software was also used for calculating the *mf_D_* value from Eq. 3 (which requires also fixing the donor lifetime). To obtain correct values, we need that intensity decays are background corrected. To do this, we estimate an average background from a non fluorescent region of interest that we subtract from the temporal histograms.

### Solutions, Cell Culture and Transfection

A first solution containing Rhodamine 6G (Rd6G) at a concentration of 5×10^−6^ M and potassium iodide (KI) at a concentration of 0.025 M was prepared (50% water and 50% ethanol). A second solution of Acridine Orange (AO) at a concentration of 5×10^−6^ M and potassium iodide (KI) at a concentration of 0.025 M was prepared (50% water and 50% ethanol). The fluorescence lifetimes of these two solutions were measured with our TD-FLIM system. We found respectively 2.54 ns for Rd6G and 1.66 ns for AO; these values are used for mimicking the lifetime of the donor alone and the lifetime of the donor when FRET occurs. The excitation and emission spectra of both solutions measured with a spectrofluorimeter (Fluorolog, Horiba Jobin Yvon) are presented in Figure S3 showing that both dyes were efficiently excited at an excitation wavelength of 488 nm. In order to simulate a FRET system with different fractions of interacting donor, we produced different mixtures of the pure solutions of Rd6G and AO: 90/10, 70/30 and 50/50. The real *f_D_* contribution for each mixture has been calculated by taking into account the pre-exponential factor coming from each pure fluorescence decay (*a* = *I/τ* where *I* corresponds to the total integral intensity and *τ* is the fluorescence lifetime), we found *f_D_* = 0.17, 0.44 and 0.65.

3T3 cells were cultured in Dulbecco’s modified Eagle’s medium containing 10% fetal bovine serum (PAA Laboratories GmbH, Pasching, Austria). The cultures were incubated at 37°C in a humidified atmosphere of 5% CO2. 3T3 cells were seeded on Mattek coverslips at a density of 2×10^5^ cells. When cells reached 70% confluence, they were transfected with a total amount of 1 µg of expression vectors (either Rac-GFP+mCherry (negative control) or Rac-GFP+PBD-mCherry [30]) using Nanofectin I (PAA). Twenty-four hours after transfection, cell medium was changed. A special DMEM-F12 medium was used to prevent auto-fluorescence (DMEM-F12 without phenol red, B12 vitamin, riboflavin and supplemented with 20 mM HEPES and L-Glutamine from PAA).

## Results

Characterizing transient interactions between dynamic proteins in living cells necessitates performing fast FRET measurements. Experimentally, this requires the ability of obtaining accurate FRET parameters with short acquisition times. According to the TD-FLIM experimental set up, two possibilities may be envisaged for shortening the acquisition time: either by reducing the number of photons *N* and/or the number of temporal channels *N_ch_*. Both issues are discussed in the next sections.

### Standard Fitting Method for Low Numbers of Photons

**Figure 3:**
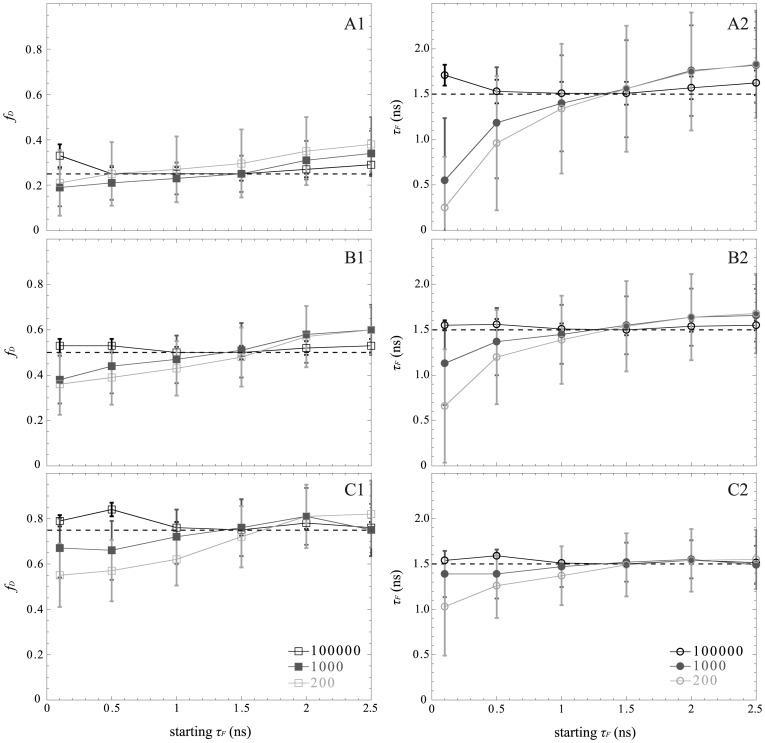
Importance of the initial conditions for the standard fitting method for three distinct total numbers of photons: 200 (in light gray), 1000 (in dark gray) and 100000 (in black). We have considered three fractions of interacting donor *f_D_*: 0.25 (A), 0.5 (B) and 0.75 (C). The minimal fractions of interacting donor are plotted in the left part and the lifetimes of the donor in presence of the acceptor in the right part. For each condition, the dotted lines represent the simulated values and the markers with error bars represent the medians and interquartile ranges of 4096 simulated TCSPC histograms (with *τ_F_* = 1.5 ns, *τ_D_* = 2.5 ns and *N_ch_* = 64 channels).

The fitting method remains the most common strategy for determining FRET parameters from TD-FLIM images [1], [29], [31]. In order to investigate the performance of this standard fitting method, specifically when the number of photons is low, we have simulated bi-exponential intensity decays (with a donor lifetime of 2.5 ns, a donor lifetime in presence of the acceptor of 1.5 ns and a fraction of interacting donors of 0.25, 0.5 and 0.75) with a constant number of temporal channels (*N_ch_* = 64) and different total numbers of photons (*N* = 100000, 1000 or 200) acquired with TCSPC technique. In order to improve the estimation of the FRET parameters (and for a fair comparison with non-fitting strategies), we have reduced the number of unknown parameters in Eq. 1 by fixing the donor lifetime to 2.5 ns. We have investigated the effect of modifying the initial parameters (*f_D_* and *τ_F_*) on the estimated FRET parameters with the standard fitting method. The results reported in Fig. 3 indicate that the FRET parameters estimated by the standard fitting method are dependent on the initial conditions when the amount of counted photons is reduced. For instance, for *N* = 200 photons, the standard fitting method is able to estimate correctly both FRET parameters (*f_D_* and *τ_F_*) if the initial conditions are: *f_D_* = 0.5 and *τ_F_* = 1.5 ns. In other words, we need to know the FRET parameters in order to estimate them correctly with the standard fitting method when the number of photons is low, which is not appropriate for automated analysis of FRET-FLIM experiments.

### Theoretical Comparison of the Non-fitting Strategies as a Function of the Number of Photons

For investigating the performance of all non-fitting strategies, we have calculated the means *µ*, and the standard deviations *σ*, of each method as a function of the number of photons *N*, based on the exhaustive work performed by Philip & Carlson [32]. For *mf_D_*, we obtain (cf Text S1)
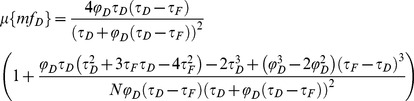
(13)

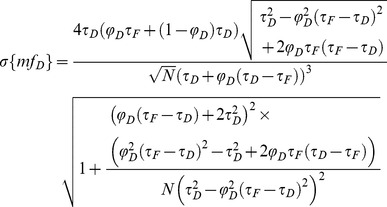
(14)


For *τ_D_* = 2.5 ns and *τ_F_* = 1.5 ns, we have plotted in Fig. 1B and C, the means and the standard deviations of *mf_D_* as a function of the number of photons *N* for three distinct fractions of interacting donor (*f_D_* = 0.25, 0.5 and 0.75). As expected, the standard deviations decrease when the number of photons increases and the means converge rapidly to the theoretical value of *mf_D_* (obtained for *N*→∞). For example, the differences between the theoretical and the calculated expectations of *mf_D_* are less than 0.05 when the number of photons *N* is more than 120 photons (for the three considered fractions of interacting donor). However, as previously explained, we emphasize the fact that these theoretical and calculated expectations of *mf_D_* underestimate the true *f_D_* values.

We proceed in the same way as previously described for calculating the mean and the standard deviation of *f_D_^P^*; we obtain
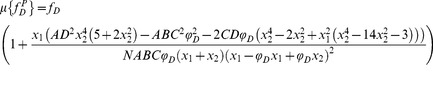
(15)

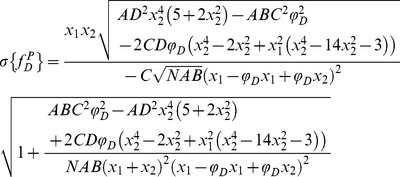
(16)where we introduce the notations



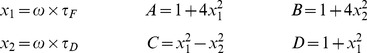
(17)For *τ_D_* = 2.5 ns and *τ_F_* = 1.5 ns, Fig. 1B and C show the variations of the means and the standard deviations of *f_D_^P^* versus the number of photons *N* for the same three fractions of interacting donors. We notice that the means converge rapidly to the theoretical values (obtained for *N*→∞) since the differences between the calculated and the theoretical expectations of *f_D_* are less than 0.05 when the number of photons is above 140. In the same way as for *mf_D_*, the standard deviations of *f_D_^P^* decrease monotonously when *N* increases and we remark that the standard deviations of *f_D_^P^* are larger than those of *mf_D_*. However, in contrast with *mf_D_* it is important to notice that the calculated expectations of *f_D_^P^* correspond to the true *f_D_* values.

We have finally investigated the performance of the analytical *f_D_^M^* when the number of photons is low. By applying the same procedure as previously described, we found the mean and the standard deviation of *f_D_^M^*

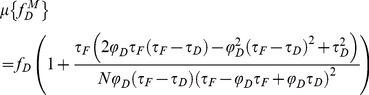
(18)

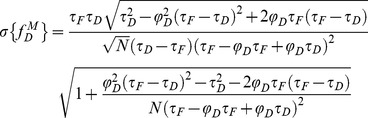
(19)


Using *τ_D_* = 2.5 ns and *τ_F_* = 1.5 ns, the variations of the means and the standard deviations of *f_D_^M^* as a function of the number of photons are represented respectively in Fig. 1B and C. For each fraction of interacting donor (*f_D_* = 0.25, 0.5 and 0.75), we show that the means converge more rapidly to the theoretical values than the polar approach. For instance, the differences between the calculated and the theoretical expectations of *f_D_* are less than 0.05 when the number of photons is above 100. The standard deviations of *f_D_^M^* are also slightly reduced in comparison with those of *f_D_^P^*, suggesting that the moments method should be more accurate than the polar approach.

### Comparison between the Non-fitting Strategies as a Function of the Number of Photons

**Figure 4:**
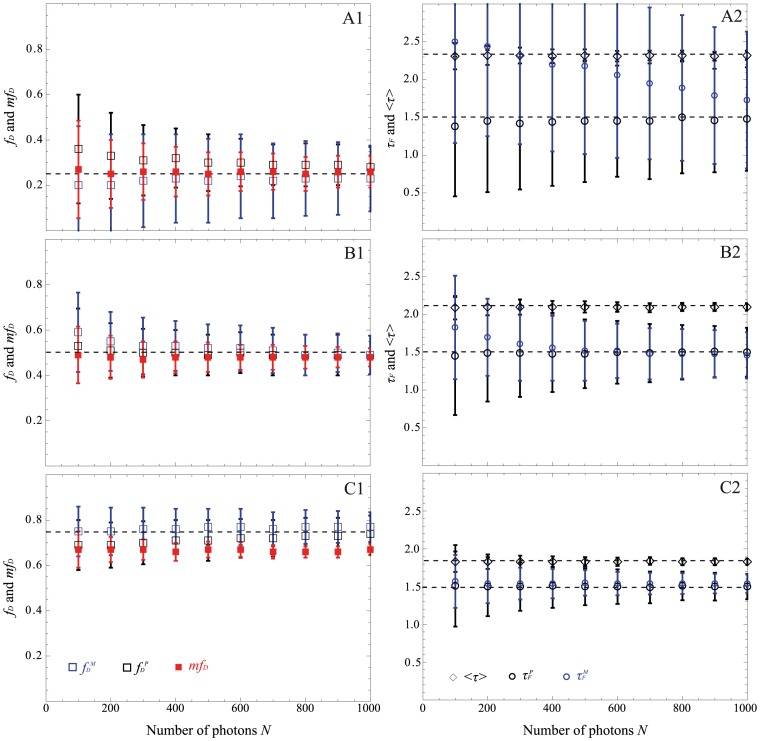
Comparison of the performance of the non-fitting strategies as a function of the total number of photons for three distinct fractions of interacting donor *f_D_*: 0.25 (A), 0.5 (B) and 0.75 (C). The polar approach is indicated in black, the moments method in blue and the *mf_D_* in red. For all methods, we have reported the estimated *f_D_* and the estimated *mf_D_* value in the left part of the figure. The estimated donor lifetime in presence of the acceptor *τ_F,_* and the estimated mean lifetime <*τ*> are reported in the right part. In all cases, medians are indicated with markers and error bars correspond to the interquartile ranges of 4096 simulated histograms whose parameters are: *τ_F_* = 1.5 ns, *τ_D_* = 2.5 ns and *N_ch_* = 64 channels (TCSPC simulations) and the simulated values are indicated in dotted lines.

In order to investigate the performance of all non-fitting methods (*mf_D,_* polar approach and moments method) when the number of photons is low, we have simulated bi-exponential intensity decays with a donor lifetime of 2.5 ns, a donor lifetime in presence of the acceptor of 1.5 ns and with distinct fractions of interacting donors 0.25, 0.5 and 0.75. We have first considered a constant number of temporal channels of 64 with different number of counted photons, varying from 100 to 1000 acquired with the TCSPC technique. The results presented in Fig. 4 indicate as expected that the calculated *mf_D_* underestimates the true *f_D_* for 0.5 and 0.75 but the difference between both values does not exceed 0.09 for all considered fractions of interacting donor. Furthermore, as anticipated from the theory when *N*≥200 photons, the polar approach gives reliable and unbiased fractions of interacting donor *f_D_^P^* and lifetime values *τ_F_^P^* with an expected increased interquartile range when the number of photons decreases. Indeed, for the same previous example (*N* = 200 photons and *f_D_* = 0.25), the difference between the estimated and the simulated *f_D_* is 0.08 and the difference between the estimated and the simulated *τ_F_* is around 50 ps. The average lifetime remains also accurate for all considered conditions. Finally, with the proposed moments method, the accuracy and precision of both FRET parameters (*f_D_^M^* and *τ_F_^M^*) are theoretically expected to be improved in comparison with the polar approach. This is the case for *f_D_* = 0.5 and 0.75 and particularly when the number of photons is high. For instance, for *N* = 1000 photons and *f_D_* = 0.75, the difference between the estimated and the simulated *f_D_* is 0.02 and the difference between the estimated and the simulated *τ_F_* is 40 ps. Furthermore, the interquartile ranges of *f_D_^M^* and *τ_F_^M^* which are respectively 0.06 and 0.12 ns are reduced compared to those of *f_D_^P^* and *τ_F_^P^* which are equal to 0.08 and 0.18 ns. However this is no longer true for *f_D_* = 0.25. In this condition, the difference between the estimated and the simulated *τ_F_* increases when *N* decreases and it becomes greater than 450 ps for the previous example (*N* = 200 and *f_D_* = 0.25). This discrepancy may be explained by the fact that the second order moment is not defined by a unique positive real root close to the limit conditions (low *N*, *N_ch_* or small *f_D_*), which lead to biased *f_D_^M^* and *τ_F_^M^* values.

From these Monte Carlo simulations, the non-fitting strategies appear robust for estimating FRET parameters (*f_D_* and *τ_F_*) when the number of photons is low (except for the moments method). However, they do not permit accurate estimation of FRET parameters when the number of photons becomes less than 200.

### Comparison between Non-fitting Strategies as a Function of the Number of Temporal Channels

If the number of photons cannot be reduced, the last solution for accelerating the acquisition time of FLIM-FRET experiments is to decrease the number of temporal channels of the time gated FLIM system (fast-FLIM prototype). Indeed, the principle of the time gated FLIM system consists of collecting (for each temporal channel) the fluorescence emitted by the sample during the selected gate width until the desired acquisition time is reached. The total acquisition time is then simply given by the product of the number of channels and the acquisition time for one channel. Consequently, a basic reduction of the number of channels allows speeding up the total acquisition time while maintaining constant the total number of photons (if the gates are contiguous and their width is adapted to the total measurement window). However this reduction of channels degrades also the accuracy of the FRET parameters estimated with non-fitting strategies (cf. Fig. S1).

The poor performance of all non-fitting strategies (*mf_D_*, polar approach and moments method) can be explained by the fact that these strategies are entirely based on the calculation of either the [*u*;*v*] coordinates or the moments *E{τ}, E{τ^2^}* which are all theoretically defined with integrals (cf. Eqs. 5, 6 and 9, 10). However, in practice, these integrals are numerically approximated because the intensity decays are constituted with a finite number of experimental points. Numerous methods are available for approximating finite integrals [33]. In this work, we use a polynomial of degree 1 as an interpolating function which means that the integrals are approximated with a sum of trapezoids. The corrected expressions obtained for all non-fitting strategies are reported in the supplementary material (texts S1, S2, S3 and S4).

**Figure 5:**
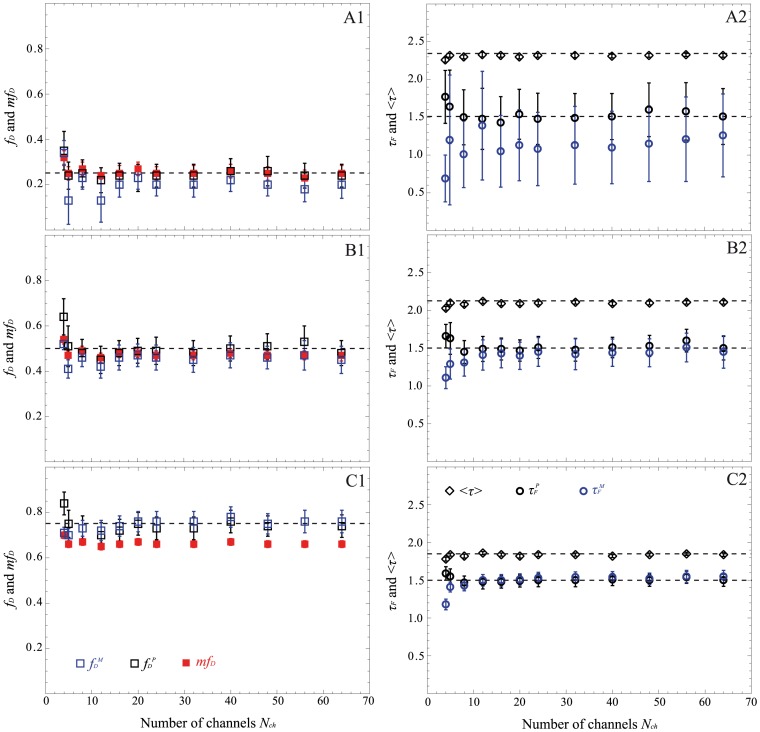
Performance of the non-fitting methods (polar approach in black, moments method in blue and *mf_D_* in red) as a function of the number of temporal channels for *N* = 1000 photons (time gated simulations). We have considered three fractions of interacting donor *f_D_*: 0.25 (A), 0.5 (B) and 0.75 (C). *τ_F_* and <*τ>* are reported in the right part whereas *f_D_^P^, f_D_^M^* and *mf_D_* are indicated in the left. For each condition, the dotted lines represent the simulated values and the markers with error bars represent the corresponding medians and interquartile ranges of 4096 simulated histograms with parameters: *τ_F_* = 1.5 ns and *τ_D_* = 2.5 ns.

For validating these corrected expressions, we have performed time gated simulations. We have simulated bi-exponential decays with the same previous parameters (*τ_D_ = *2.5 ns and *f_D_* = 0.25, 0.5 and 0.75) and we have considered a fixed number of photons (*N* = 1000 photons corresponding to 8500 grey levels in our system) and distinct number of contiguous gates varying from 4 to 64. The FRET parameters calculated from the corrected expressions are reported in Fig. 5 for all non-fitting strategies. With our corrected expressions, both the *mf_D_* and the polar approach allow correct estimation of the FRET parameters for all fractions of interacting donor. For instance, the difference between the calculated and the simulated *f_D_* values is indeed less than 0.05 and the difference between the calculated and the simulated *τ_F_* is less than 140 ps for all numbers of temporal channels (except for *N_ch_* = 4). Furthermore, the difference between the calculated *mf_D_* and the simulated *f_D_* values is always less than 0.1 for all fractions of interacting donors, even if the number of temporal channels is as low as 4, confirming the fact that this non-fitting strategy is well adapted for estimating FRET parameters in fast-FLIM-FRET experiments.

Concerning the moments method, its domain of validity is reduced. As previously described, when *f_D_* = 0.25, the difference between the calculated *f_D_^M^* and the simulated *f_D_* values can reach 0.12 and the recovered *τ_F_^M^* is not correct. For instance, the difference between the calculated *τ_F_^M^* and the simulated *τ_F_* can exceed 0.8 ns, which is not acceptable.

**Figure 6:**
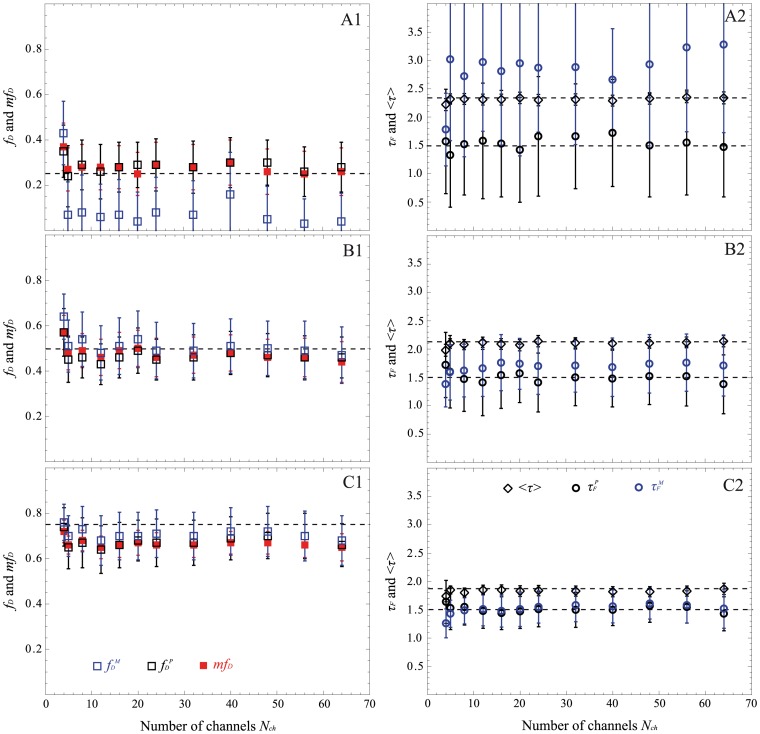
Performance of the non-fitting methods (polar approach in black, moments method in blue and *mf_D_* in red) as a function of the number of temporal channels for *N* = 200 photons acquired with time gated system. Three fractions of interacting donor *f_D_* are considered: 0.25 (A), 0.5 (B) and 0.75 (C). We have indicated the fraction of interacting donor and the *mf_D_* in the left part; the donor lifetime in presence of the acceptor *τ_F_* and the mean lifetime <*τ>* are in the right part. In all graphs, the dotted lines represent the simulated values and the markers with error bars represent the corresponding medians and interquartile ranges of 4096 simulated histograms whose parameters are: *τ_F_* = 1.5 ns and *τ_D_* = 2.5 ns.

To complete this investigation and to validate the robustness of both polar approach and *mf_D_*, additional Monte Carlo simulations were carried out in the same way (with contiguous gates varying from 4 to 64) by fixing now the number of photons to 200 (corresponding to 1700 grey levels in our time gated system). The results obtained with all non-fitting strategies are presented in Fig. 6. As expected, the moments method is not valid when *f_D_* = 0.25 whatever the number of temporal channels is (the difference between the calculated *f_D_^M^* and the simulated *f_D_* value can reach 0.22 and the difference between the calculated *τ_F_^M^* and the simulated *τ_F_* is more than 1.78 ns). But surprisingly, when the number of gates is greater than 4, the moments method is reasonably valid for *f_D_* = 0.5 and almost perfect when *f_D_* = 0.75. The domain of validity of the moments method seems to be more affected by the *f_D_* value than by the number of detected photons, even if the standard deviation increases when the number of photons decreases. Concerning the other non-fitting strategies (polar approach and *mf_D_*), they stay accurate even with only 4 gates and 200 photons. For instance, the difference between the calculated and the simulated *f_D_* values is less than 0.1 and the difference between the calculated and the simulated *τ_F_* is less than 220 ps for all number of temporal channels (except for *N_ch_* = 4). With *mf_D_*, the difference between the calculated *mf_D_* and the simulated *f_D_* values is always less than 0.12. Furthermore, as predicted by the theory, we note that the standard deviations of *mf_D_* are reduced in comparison with those of the polar approach. For example, for *N_ch_* = 8 and *f_D_* = 0.75, the interquartile range of *mf_D_* (iqr = 0.04) is less than that of *f_D_^P^* which is equal to 0.11.

### Comparison between Non-fitting Strategies on Experimental FLIM Measurements of Fluorescent Solutions using Fast-FLIM Prototype

**Figure 7:**
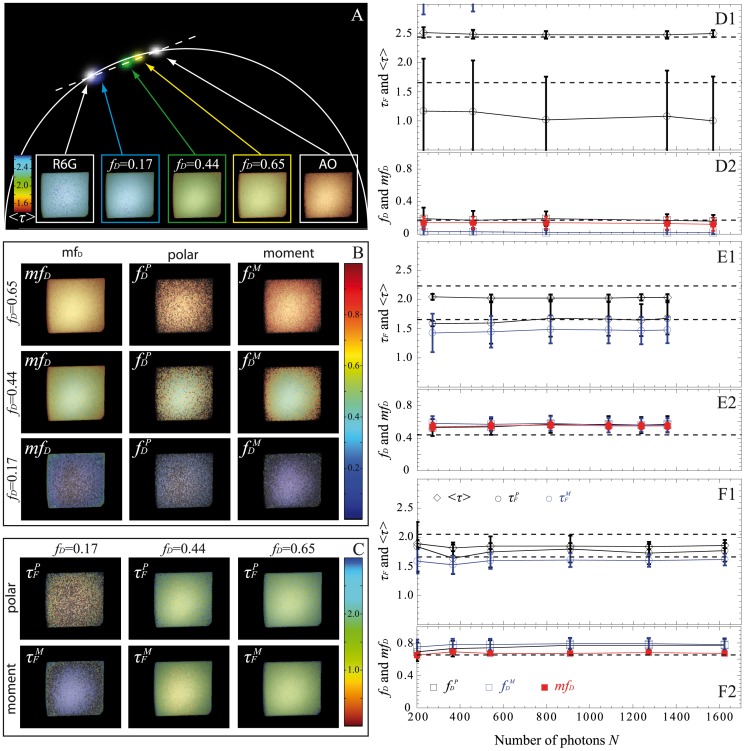
Experimental FLIM measurements on fluorescent solutions with our fast-FLIM prototype. Lifetime images of Rd6G alone, AO alone and of three mixtures of Rd6G and AO with theoretical fractions of interacting donor of 0.17, 0.44 and 0.65 were acquired with *N*≈1500 photons. The polar plot and the mean lifetime images of each solution are represented in (A). All spots corresponding to the mixtures in the polar plot are well localized on a line connecting pure Rh6G and pure AO. We have also calculated *f_D_^P^*, *f_D_^M^* and *mf_D_* for the three mixtures and the corresponding images are indicated in (B). We show in (C) the images of donor lifetime in presence of the acceptor estimated with the polar approach and the moment method. We have also performed FLIM acquisitions with different numbers of detected photons for each fraction of interacting donor: 0.17 (D), 0.44 (E) and 0.65 (F); the corresponding plots of *f_D_^P^*, *f_D_^M^*, *mf_D,_ τ_F_^P^* and *τ_F_^M^* as a function of *N* are reported in the right part. For all graphs, markers with error bars represent the medians and interquartile ranges of FLIM images. The theoretical *f_D_* and *τ_F_* are indicated in dotted lines.

In order to investigate the validity of the non-fitting strategies on experimental data, we have performed FLIM measurements of fluorophores solutions with our fast-FLIM prototype presented in Fig. 2. This time gated prototype was designed for fast acquisition of FLIM by using five gates of 2.25 ns width. We prepared three mixtures of two fluorophores, Rd6G and AO in the presence of KI in order to mimic the simulated lifetime values (see Material and Methods for details). We have acquired several FLIM images with different numbers of detected photons per pixel, *N*, varying from 200 to about 1600 (determined from S-factor quantification, see Fig. S2). These FLIM images were analyzed with all non-fitting strategies: *mf_D_,* polar approach and moments method. The results are summarized in Fig. 7. Firstly, it has to be noted that the number of detected photons, *N*, does not influence the accuracy of the FRET parameters estimated with the non-fitting strategies. Secondly, when *f_D_* = 0.17, the moments method is not valid (values of *τ_F_^M^* out of range). Even with the polar approach, *τ_F_^P^* is not accurate and the standard deviation is large. This could be easily explained in the graphical representation of the polar plot because the two dots corresponding respectively to the donor alone (Rd6G in white) and *f_D_* = 0.17 (in blue) are close, meaning that the line construction with the interception of the semi-circle for recovering *τ_F_* is not precise. Concerning the fraction of interacting donor, both strategies (*mf_D_* and polar approach) correctly estimate this parameter, which is not the case with the moments method (unacceptable underestimation of the *f_D_* value). Thirdly, for *f_D_* = 0.44 and 0.65, we can notice that the *f_D_* values are overestimated with the three non-fitting strategies but this discrepancy would probably come from the calculation of the theoretical *f_D_* of the fluorescent solutions. This hypothesis is in agreement with the diminution of the estimated mean lifetime in comparison with the theoretical one shown in panels B1 and C1 of Fig. 7 since it is well known that the mean lifetime is a robust parameter [14], [34]. Excluding this difference, all non-fitting strategies are very robust for recovering *τ_F_* and *f_D_* even when using only 5 gates which was not evident from the simulated results of the moments method (5 is sufficient whereas 4 was not enough, see Fig. 5). Finally, we note that the standard deviations of the different non-fitting strategies are coherent with the theoretical predictions of Fig. 1. As expected, *mf_D_* is the less noisy strategy and the moments method (when it is valid) exhibits a smaller error deviation than the polar approach (Panels E and F in Fig. 7).

### Spatio-temporal Quantification of Protein-protein Interactions in Living Cell Using Fast-FLIM Prototype and Non-fitting Approaches

**Figure 8:**
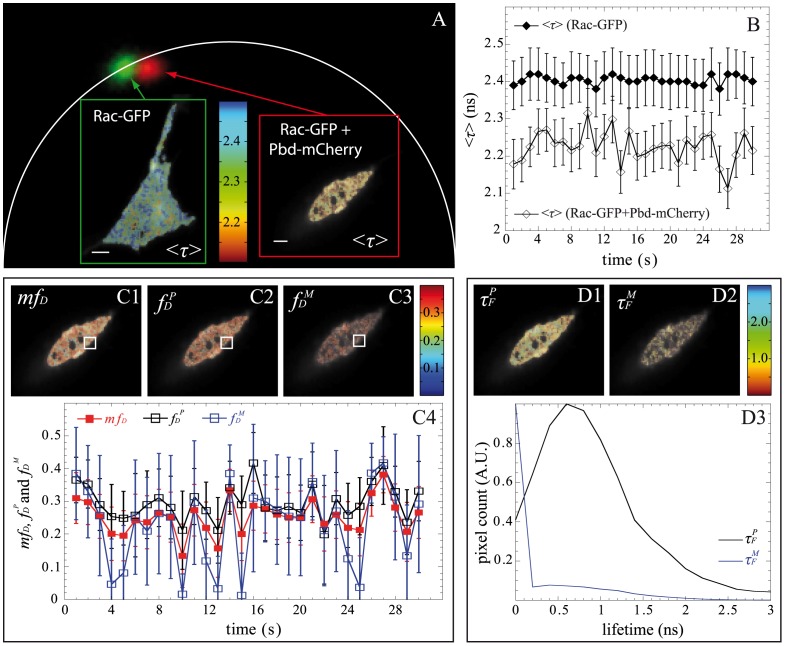
Experimental FLIM measurements with our fast-FLIM prototype for quantifying G protein activation in living cells. The polar plots of a 3T3 cell co-expressing Rac-GFP and PBD-mCherry (with *N_mean_* = 850 photons) and of a cell co-expressing Rac-GFP+mCherry (with *N_mean_* = 830 photons) as a reference (negative control) are shown in (A). The shift between the two spots is the proof of lifetime modification which is also clearly visible in the mean lifetime images <*τ*> (scale bar: 10 µm). We have plotted in (B) the temporal variations of the mean lifetime for each cell. We have also calculated *f_D_^P^*, *f_D_^M^* and *mf_D_* of the cell co-expressing Rac-GFP and PBD-mCherry and the images are reported respectively in (C1), (C2) and (C3). The evolution of each parameter calculated in the white region of interest is also plotted in (C4). We finally show the images of *τ_F_^P^* and *τ_F_^M^* in (D1) and (D2) and the corresponding lifetime distributions in (D3).

To demonstrate the ability of the non-fitting strategies to probe protein-protein interactions in live cells by FRET with the fast-FLIM prototype, we applied them to the quantification of G protein activation in 3T3 cells [15], [24]. The Rho family of small GTPases regulates cell shape and motility. When Rac is in its active form (GTP bound state), it interacts with effectors that affect actin polymerization. The PBD assay allows detection of the active form of Rac (Fig. S4) and in our case, PBD-mCherry was expressed throughout the cell (meaning that no particular sub-localization was found). FLIM images of a 3T3 cell co-expressing Rac-GFP and PBD-mCherry and a cell co-expressing Rac-GFP+mCherry as a reference (negative control) were acquired with our fast-FLIM prototype. From the S-factor, we deduced that the detected mean numbers of photons were respectively 830 and 850 photons for the reference and the cell co-expressing Rac-GFP and PBD-mCherry. The FLIM images were then analyzed with all non-fitting strategies (*mf_D_*, polar approach and moments method) and the results are reported in Fig. 8. Both, the polar plot (Fig. 8A) and the corrected mean lifetime (Fig. 8B) show a modification of the lifetime caused by FRET. Fig. 8B shows a global mean lifetime decrease of the cell co-expressing Rac-GFP and PBD-mCherry (<*τ*> = 2.21+/−0.06) relative to the control cell expressing Rac-GFP only in the presence of diffusing mCherry (<*τ*> = 2.40+/−0.07). This diminution was also observed in the whole population of analyzed cells; we found a mean lifetime <*τ*> = 2.42+/−0.03 ns (n = 10) for the negative control cells which decreases to 2.37+/−0.02 ns (n = 10) for the co-expressing cells (Fig. S4F). The fast-FLIM prototype offers the possibility of acquiring data very rapidly (in this case, 1 FLIM image per sec). Our aim is to analyze the spatio-temporal evolution of Rac activation for a small region of interest. We have then calculated the fraction of interacting donors with all non-fitting approaches (cf. Fig. 8C). Fig. 8C4 shows that *mf_D_*_,_ *f_D_^P^* and *f_D_^M^* have similar temporal oscillations which correspond to transient transitions of Rac between GDP and GTP forms. These oscillations indicate real Rac1-PBD interactions since such variations of *mf_D_*_,_ *f_D_^P^* and *f_D_^M^* are not present in cell co-expressing Rac-GFP+mCherry (cf. Fig. S5). Note that the values of *mf_D_* and *f_D_^P^* are almost similar since for the typical range of lifetimes of the fluorescent proteins (around 2.5 ns) *τ_F_* lifetimes are close to <*τ*>/2 which implies that *mf_D_* = *f_D_*. We note also that *f_D_^M^* presents significant lower values than *mf_D_* or *f_D_^P^* when the fraction of donor in interaction is lower than 0.25. This result is in agreement with our previous results obtained from Monte Carlo simulations and experimental measurements on fluorescent solutions. This behavior is also visible in Fig. 8C3 because *f_D_^M^* is non null only when the fraction of interacting donor (*mf_D_* or *f_D_^P^*) is greater than 0.2; otherwise the pixel of *f_D_^M^* image is black. We have represented in Fig. 8D, the lifetime images of *τ_F_^P^* and *τ_F_^M^*. The *τ_F_^P^* image is well resolved and its distribution is centered around 0.7 ns. Concerning the *τ_F_^M^* image, the pixels can be divided into two populations: the pixels in black correspond to *f_D_^M^* lower than 0.2 (out of the domain of validity of the moments method) and the others correspond to *f_D_^M^* higher than 0.2 and consequently correct fluorescence lifetime values. These results confirm that the combination of our fast-FLIM prototype with direct non-fitting methods (which are easily automated) allows quantifying correctly the spatiotemporal FRET parameters for Rac activation in living cells and more generally for protein-protein interactions.

## Discussion

We have explored theoretically, computationally and experimentally the performances of both fitting and non-fitting methods when performing FLIM-FRET experiments. We have first shown that the correct interpretation of FLIM data necessitates a high amount of photons (cf. Fig. 3) when applying standard fitting procedures even for the simplest FRET system with two populations (donor and FRET species) that should be well fitted with a double exponential model [14], [25], [28]. Therefore extremely long acquisition times (several minutes) are required to collect enough photons per pixel. If the number of counted photons is too low, the results estimated from the standard fitting method depend on the initial conditions, and information about the wanted FRET parameters (*τ_D_*, *τ_F_* and *f_D_*) must be known beforehand for obtaining accurate values. Several techniques have been developed for simplifying the problem [28]; like for instance global analysis [8], [35] which consists in linking FRET parameters and considering globally many fluorescence decays coming from the same image (or from other experiments). However this necessitates making the assumption that all pixels have the same FRET parameters, this also requires expertise in applying the right mathematical model [1] and the computing time is not negligible which is not compatible with online data analysis and makes it more difficult for quantifying protein-protein interactions in living cells, especially in dynamic systems.

In this work, we have considered three non-fitting strategies: the polar approach, the moments method and the minimal fraction of interacting donor. Based on Monte Carlo simulations and experimental measurements, we have evaluated the performance of each strategy as a function of (i) the photon budget (cf. Fig. 4) and (ii) the number of temporal channels (cf. Fig. 5 and 6) for various fractions of interacting donor (*f_D_*). Even if we consider a limited number of specific FRET conditions, the covered range is well representative of the usual FRET parameters encountered in FRET experiments [34], [36], [37]; this implies that our results could be easily extrapolated to all FRET experiments.

From both the simulated and experimental data, we can infer that the polar approach is the more robust method for correctly characterizing a 2 populations system consisting of donor and FRET species. Indeed, the accurate values of *f_D_^P^* and *τ_F_^P^* can be successfully obtained with only 4 gates even if the amount of signal is low and the amount of interacting proteins is small. To achieve such accuracy, it is essential to take into account both the finite width of the temporal channels and the finite width of the total measurement window. In this work, the exponential decays are approximated with simple linear functions and the resulting integral computation error is successfully compensated even if the number of temporal channels is as low as 4 [17].

The moments method that we have introduced in this work turned out to be unreliable when the fraction of interacting donor is low and/or when the number of either counted photons or temporal channels is low (see Fig. 6, the estimated parameters can be largely different from the simulated values) and it gives unacceptable values when *f_D_* is less than 0.2 and/or when the number of temporal channels is equal to 4. This can be explained by the fact that the second order moment is theoretically the only positive real root of a polynomial of high degree. However, close to the limit conditions (low *N*, *N_ch_* or small *f_D_*), several real positive roots exist which lead to biased *f_D_^M^* and *τ_F_^M^* values.

Curiously enough, this is not the case with the first order moment and consequently the *mf_D_* calculation which appears reliable in all possible scenarios when applying to single exponential donors. It must be highlighted that the domain of validity of *mf_D_* is dependent on the lifetime of the donor in presence of the acceptor. As seen in Fig. 1 and 8 and explained in a previous work [14], when *τ_F_* is far from half of the mean lifetime (<*τ*>/2), the minimal fraction of interacting donor is very different of the true *f_D_*. However, in most biological systems that we have tested (Histone H4 acetylation [14], Amphiphysin-NWASP interaction [34], Rac-PBD interaction [38], and that we have found in the literature [39], [40]), the lifetime *τ_F_* is usually close to <*τ*>/2 which explains why the *mf_D_* is a robust approach in living cells. Another benefit of *mf_D_* is its small standard deviation in comparison with the polar approach and the moments method. As anticipated from the theory (Fig. 1) and corroborated with both Monte Carlo simulations (Fig. 4–6) and experimental results (Fig. 7), *mf_D_* is the most precise strategy and the error of the moments method (when it is valid) is smaller than those of the polar approach.

In this study, we have considered the simplest FRET system consisting of donor species whose fluorescence decays are mono-exponential. However, we emphasize the fact that the non-fitting strategies that we have presented here are not limited to this simple case. It has indeed been already demonstrated that the *mf_D_* could be successfully calculated with multiple lifetime donor [14]. Concerning the polar approach and the moments method, a theoretical formulation of this situation is under investigation.

In this work, the experimental FLIM measurements were performed with a time-gated system. We show that the combination of our fast-FLIM prototype with non-fitting based analysis strategies allows us to investigate the spatio-temporal regulation of Rac activation in live cells at a frequency up to 1 Hz which becomes interesting in terms of the cell regulation of such biochemical signal. Additionally, the use of these strategies for FLIM image analysis can be directly implemented on-line on a standard computer and thus are very powerful for quantifying automatically the spatio-temporal regulation of protein-protein interactions and biochemical activities in living cells by FRET. Moreover, we emphasize on the fact that the non-fitting strategies introduced here are also easily applicable with all existing TD FLIM techniques including those using a streak camera [41], [42] or a time-correlated single photon counting system.

## Supporting Information

Figure S1 **Performance of the non-fitting methods as a function of the number of temporal channels for** ***N*** **= 200 photons acquired with time gated system.** Simulations were performed with various fractions of interacting donor *f_D_*: 0.25 (A), 0.5 (B) and 0.75 (C). The fraction of interacting donor *f_D_* and *mf_D_* are represented in left part; the donor lifetime in presence of the acceptor *τ_F_* and the mean lifetime <*τ*> are plotted in the right part. If we do not compensate for the number of temporal channels and the finite measurement width, all non-fitting methods: *mf_D_* (in red), polar approach (in black) and moments methods (in blue) do not satisfactorily estimate *f_D_*, *τ_F_*, or <*τ*>. For instance for *N_ch_*≤16 (and *f_D_* = 0.25), the differences between the calculated *mf_D_* and the simulated *f_D_* values are superior to 0.2, the differences between the calculated *τ_F_^P^* and the simulated *τ_F_* exceed 500 ps with the polar approach and the differences between the calculated *τ_F_^M^* and the simulated *τ_F_* exceed 1500 ps with the moments method. The markers correspond to the median of each estimated parameter and the error bars correspond to the interquartile ranges. All Monte Carlo simulations were performed with: *τ_F_* = 1.5 ns, *τ_D_* = 2.5 ns and *N* = 200 photons.
(TIF)

Figure S2 **S factor.** The S factor was calculated to convert the arbitrary units of fluorescence intensity into number of photons. We have acquired the fluorescence signal emitted by a defined region of interest of a fluorescent slide from Chroma Technologies (Germany). We have performed several experiments with various exposure times and we plotted the variance of these experiments against the intensity. The experimental points are fitted with a linear function which is indicated in grey line. The slope of this line was found to be 8.5 grey level/photon.
(TIF)

Figure S3 **Acridine Orange and Rhodamine 6G excitation and emission spectra.** Four experiments were carried out with a spectrofluorimeter (Fluorolog, Horiba Jobin-Yvon, France) on the 50/50 mixture of Acridine Orange (black stripped line) and Rhodamin 6G (red stripped line) in order to obtain both the excitation spectra and the respective emission spectra (black and red solid lines). We have also shown the excitation filter band that was employed with our fast-FLIM prototype (480–490 nm) and the corresponding emission filter (500–550 nm).
(TIF)

Figure S4 **Quantitative FRET imaging with fast-FLIM to probe Rac GTPase activity.** (A) Cartoon describing the Rac-PBD assay. The co-expression of PBD-mCherry together with Rac-eGFP allows for the detection of GTPase activity since a conformational change occurs during the GDP/GTP interchange, which reduces the distance between the two fluorescent proteins and consequently FRET occurs. In this situation, the fluorescence decay of the GFP is faster compared to the fluorescence decay alone. (B) Two representative cells co-expressing Rac-GFP+mCherry alone on one hand (first row) and Rac-GFP+PBD-mCherry on the other hand (bottom row). The images of intensity (first column), non-corrected average lifetime (second row) and *mf_D_* (third and last row) are presented. The pseudo-color bar of the FLIM images clearly shows a general average lifetime diminution (from blue to green, or from 2.45+/−0.02 ns to 2.30+/−0.07 ns). The *mf_D_* approach shows an increase of the minimal fraction of interacting donor for this cell (from 0.01+/−0.02 to 0.19+/−0.04). (C) The fluorescence intensities as a function of time for the two regions of interest are shown; in this case no photo-bleaching was observed during the time-lapse given the fact that both intensity traces are steady over time. (D) The non-corrected average lifetime was calculated for each image of the time-lapse, and the mean values coming from the ROI depicted in (B) are plotted as a function of time. (E) The *mf_D_* values were also calculated for the same ROIs and their evolution as a function of time is shown. (F) The lifetime diminution (FRET) was also calculated for a population of cells (n = 10), and the average lifetimes coming from the mean value of all pixels for each experiments are shown. There is a global diminution in the non-corrected average lifetime that goes from 2.42+/−0.03 ns to 2.37+/−0.02 ns.
(TIF)

Figure S5 **Calculations of** ***mf_D_, f_D_^P^*** **and** ***f_D_^M^*** **in a cell co-expressing Rac-GFP+mCherry (negative control).** We have reported the corresponding images of *mf_D_, f_D_^P^* and *f_D_^M^* in the upper part of the figure. The evolution of each parameter calculated in the white region of interest is also plotted in the lower part. Markers with error bars represent the medians and interquartile ranges of each parameter.
(TIF)

Text S1 **Theoretical calculations of the means and the standard deviations as a function of the number of photons.**
(DOC)

Text S2 **Corrected expressions of the non fitting approaches.**
(DOC)

Text S3 **Corrected expression of the mean lifetime.**
(DOC)

Text S4 **Corrected expression of the second moment.**
(DOC)
