# Correction: 827Spatio-Temporal Quantification of FRET in Living Cells by Fast Time-Domain FLIM: A Comparative Study of Non-Fitting Methods

**DOI:** 10.1371/annotation/75f99136-45ec-4078-932e-07ed1242a73e

**Published:** 2013-08-02

**Authors:** Aymeric Leray, Sergi Padilla-Parra, Julien Roul, Laurent Héliot, Marc Tramier

There was an error in the title. The correct title is: Spatio-Temporal Quantification of FRET in Living Cells by Fast Time-Domain FLIM: A Comparative Study of Non-Fitting Methods.

The correct citation is: Leray A, Padilla-Parra S, Roul J, Héliot L, Tramier M (2013) Spatio-Temporal Quantification of FRET in Living Cells by Fast Time-Domain FLIM: A Comparative Study of Non-Fitting Methods. PLoS ONE 8(7): e69335. doi:10.1371/journal.pone.0069335. 

