# Supplementary figures and images for: 827Spatio-Temporal Quantification of FRET in Living Cells by Fast Time-Domain FLIM: A Comparative Study of Non-Fitting Methods
Source: PLoS One. 2013 Jul 18;8(7):e69335. doi: 10.1371/journal.pone.0069335 (PMC3715500; doi:10.1371/journal.pone.0069335)

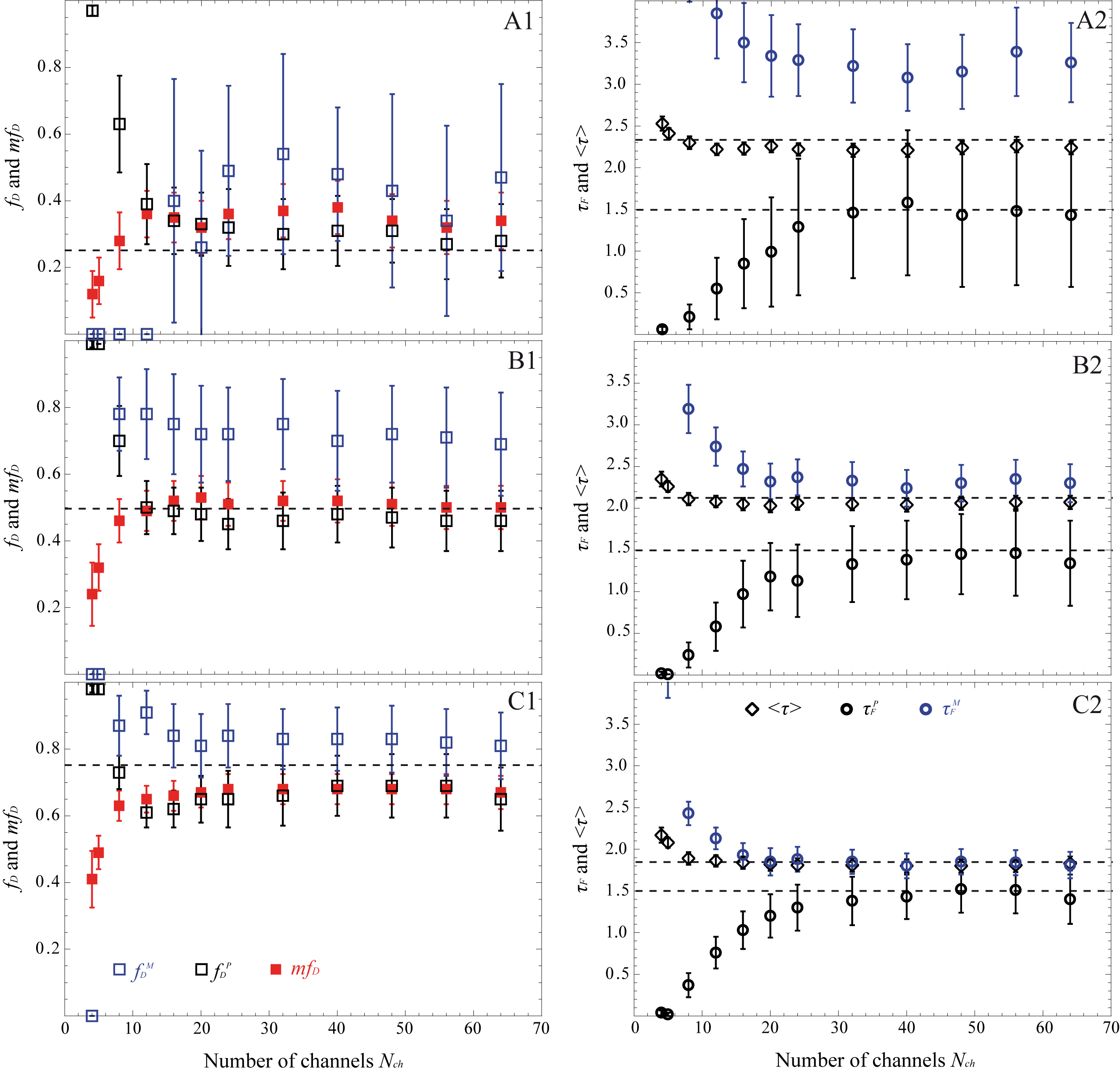

Supplement: Figure S1 — Performance of the non-fitting methods as a function of the number of temporal channels for N = 200 photons acquired with time gated system. Simulations were performed with various fractions of interacting donor fD: 0.25 (A), 0.5 (B) and 0.75 (C). The fraction of interacting donor fD and mfD are represented in left part; the donor lifetime in presence of the acceptor τF and the mean lifetime <τ> are plotted in the right part. If we do not compensate for the number of temporal channels and the finite measurement width, all non-fitting methods: mfD (in red), polar approach (in black) and moments methods (in blue) do not satisfactorily estimate fD, τF, or <τ>. For instance for Nch≤16 (and fD = 0.25), the differences between the calculated mfD and the simulated fD values are superior to 0.2, the differences between the calculated τFP and the simulated τF exceed 500 ps with the polar approach and the differences between the calculated τFM and the simulated τF exceed 1500 ps with the moments method. The markers correspond to the median of each estimated parameter and the error bars correspond to the interquartile ranges. All Monte Carlo simulations were performed with: τF = 1.5 ns, τD = 2.5 ns and N = 200 photons. (TIF) [file pone.0069335.s001.tif]

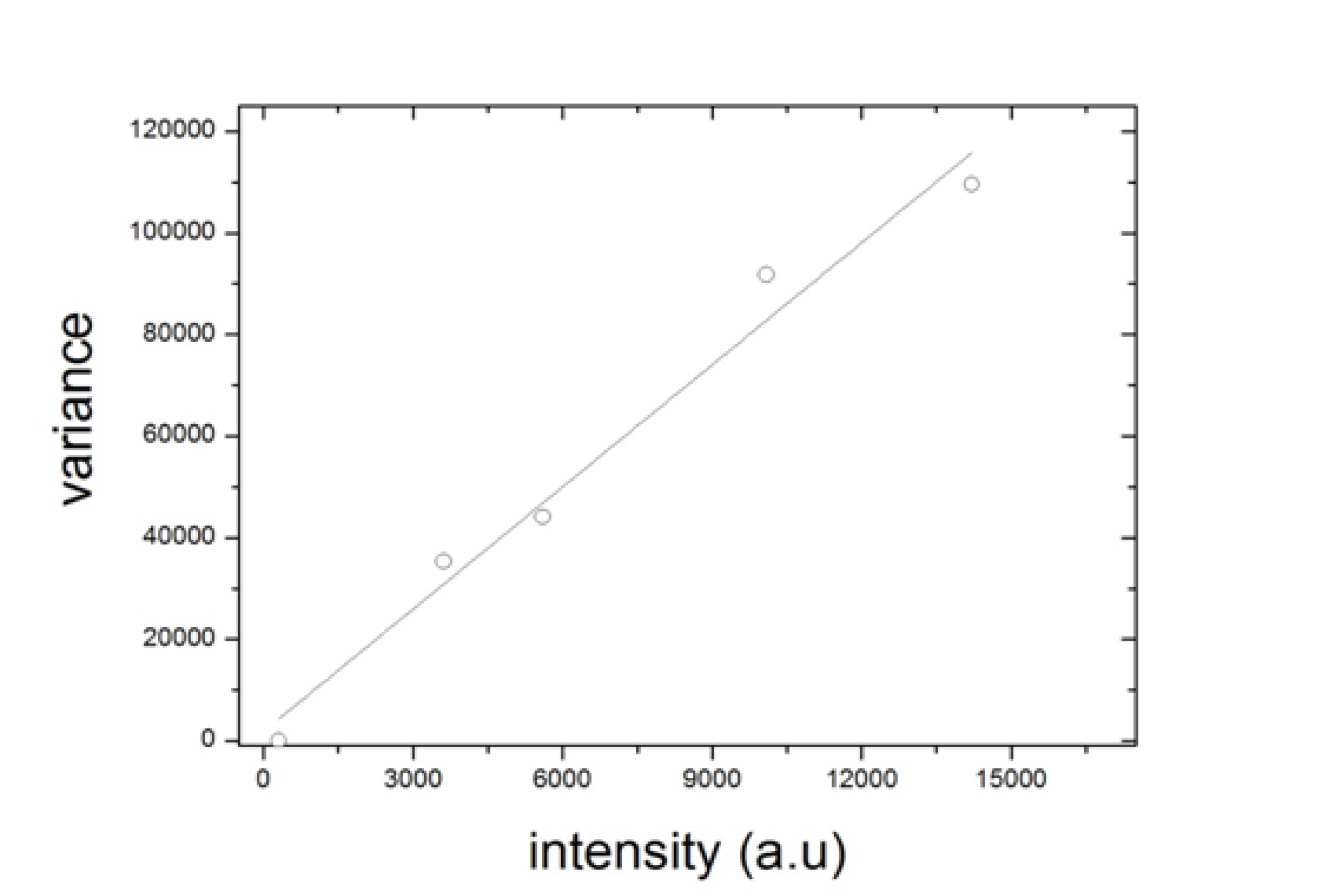

Supplement: Figure S2 — S factor. The S factor was calculated to convert the arbitrary units of fluorescence intensity into number of photons. We have acquired the fluorescence signal emitted by a defined region of interest of a fluorescent slide from Chroma Technologies (Germany). We have performed several experiments with various exposure times and we plotted the variance of these experiments against the intensity. The experimental points are fitted with a linear function which is indicated in grey line. The slope of this line was found to be 8.5 grey level/photon. (TIF) [file pone.0069335.s002.tif]

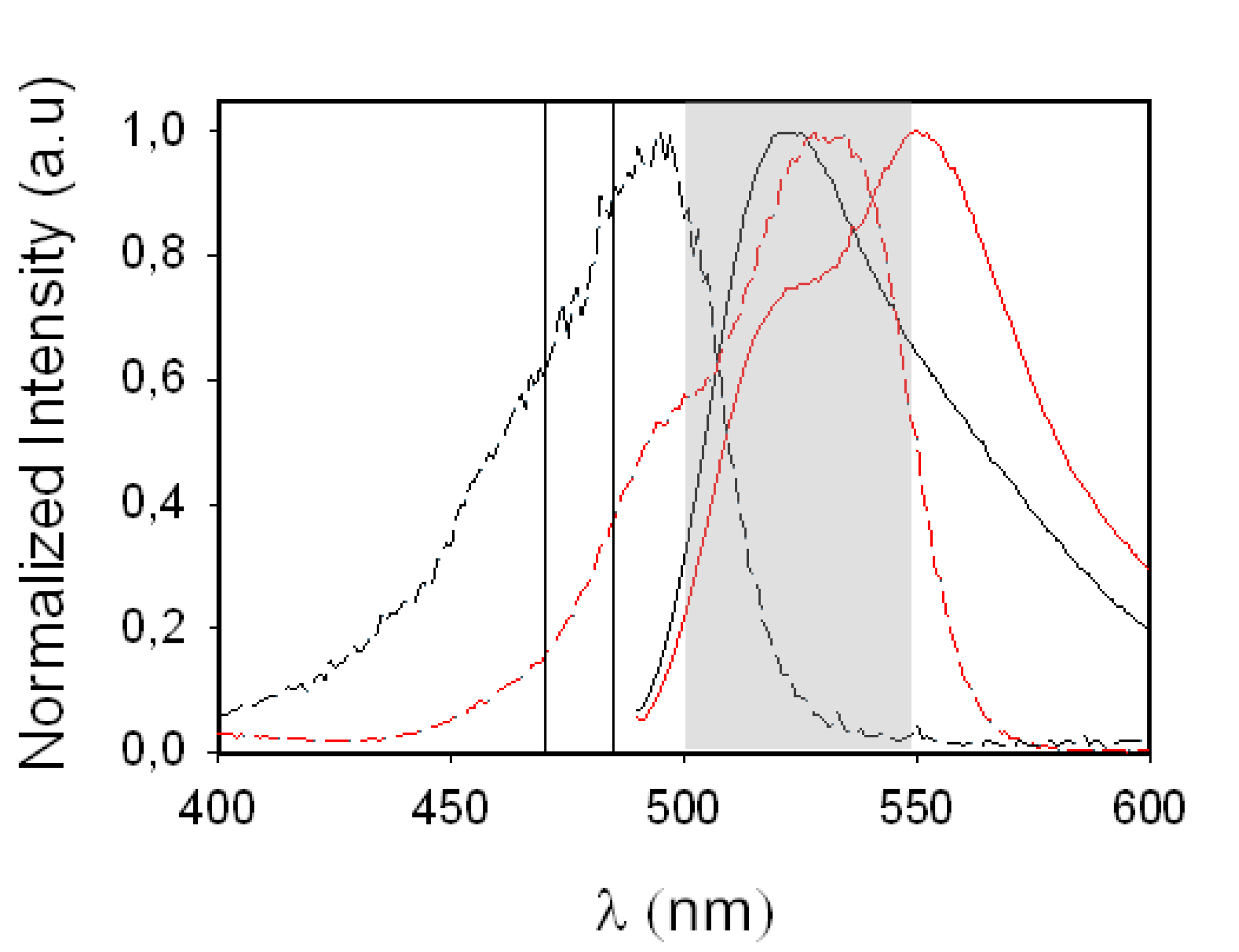

Supplement: Figure S3 — Acridine Orange and Rhodamine 6G excitation and emission spectra. Four experiments were carried out with a spectrofluorimeter (Fluorolog, Horiba Jobin-Yvon, France) on the 50/50 mixture of Acridine Orange (black stripped line) and Rhodamin 6G (red stripped line) in order to obtain both the excitation spectra and the respective emission spectra (black and red solid lines). We have also shown the excitation filter band that was employed with our fast-FLIM prototype (480–490 nm) and the corresponding emission filter (500–550 nm). (TIF) [file pone.0069335.s003.tif]

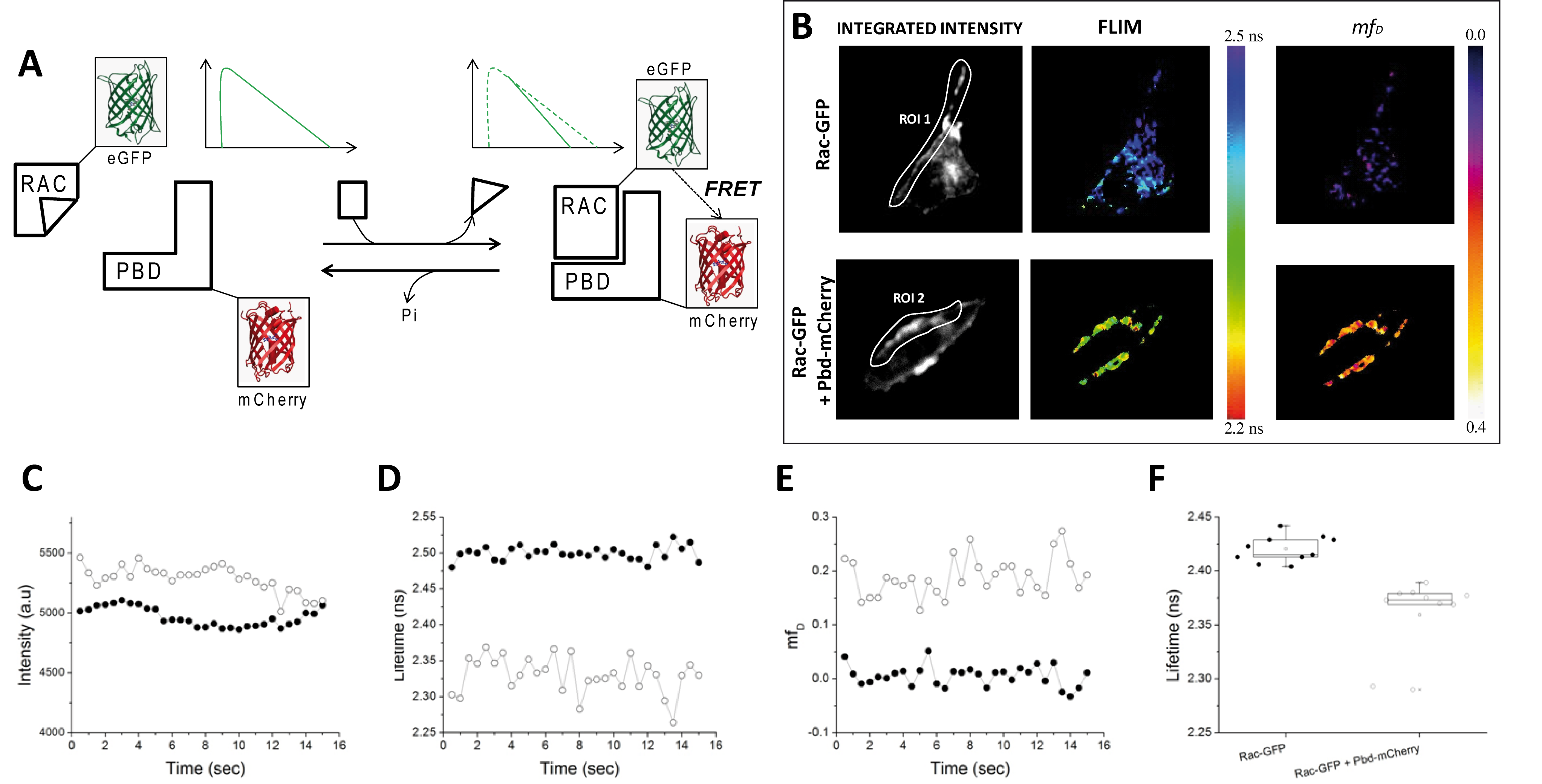

Supplement: Figure S4 — Quantitative FRET imaging with fast-FLIM to probe Rac GTPase activity. (A) Cartoon describing the Rac-PBD assay. The co-expression of PBD-mCherry together with Rac-eGFP allows for the detection of GTPase activity since a conformational change occurs during the GDP/GTP interchange, which reduces the distance between the two fluorescent proteins and consequently FRET occurs. In this situation, the fluorescence decay of the GFP is faster compared to the fluorescence decay alone. (B) Two representative cells co-expressing Rac-GFP+mCherry alone on one hand (first row) and Rac-GFP+PBD-mCherry on the other hand (bottom row). The images of intensity (first column), non-corrected average lifetime (second row) and mfD (third and last row) are presented. The pseudo-color bar of the FLIM images clearly shows a general average lifetime diminution (from blue to green, or from 2.45+/−0.02 ns to 2.30+/−0.07 ns). The mfD approach shows an increase of the minimal fraction of interacting donor for this cell (from 0.01+/−0.02 to 0.19+/−0.04). (C) The fluorescence intensities as a function of time for the two regions of interest are shown; in this case no photo-bleaching was observed during the time-lapse given the fact that both intensity traces are steady over time. (D) The non-corrected average lifetime was calculated for each image of the time-lapse, and the mean values coming from the ROI depicted in (B) are plotted as a function of time. (E) The mfD values were also calculated for the same ROIs and their evolution as a function of time is shown. (F) The lifetime diminution (FRET) was also calculated for a population of cells (n = 10), and the average lifetimes coming from the mean value of all pixels for each experiments are shown. There is a global diminution in the non-corrected average lifetime that goes from 2.42+/−0.03 ns to 2.37+/−0.02 ns. (TIF) [file pone.0069335.s004.tif]

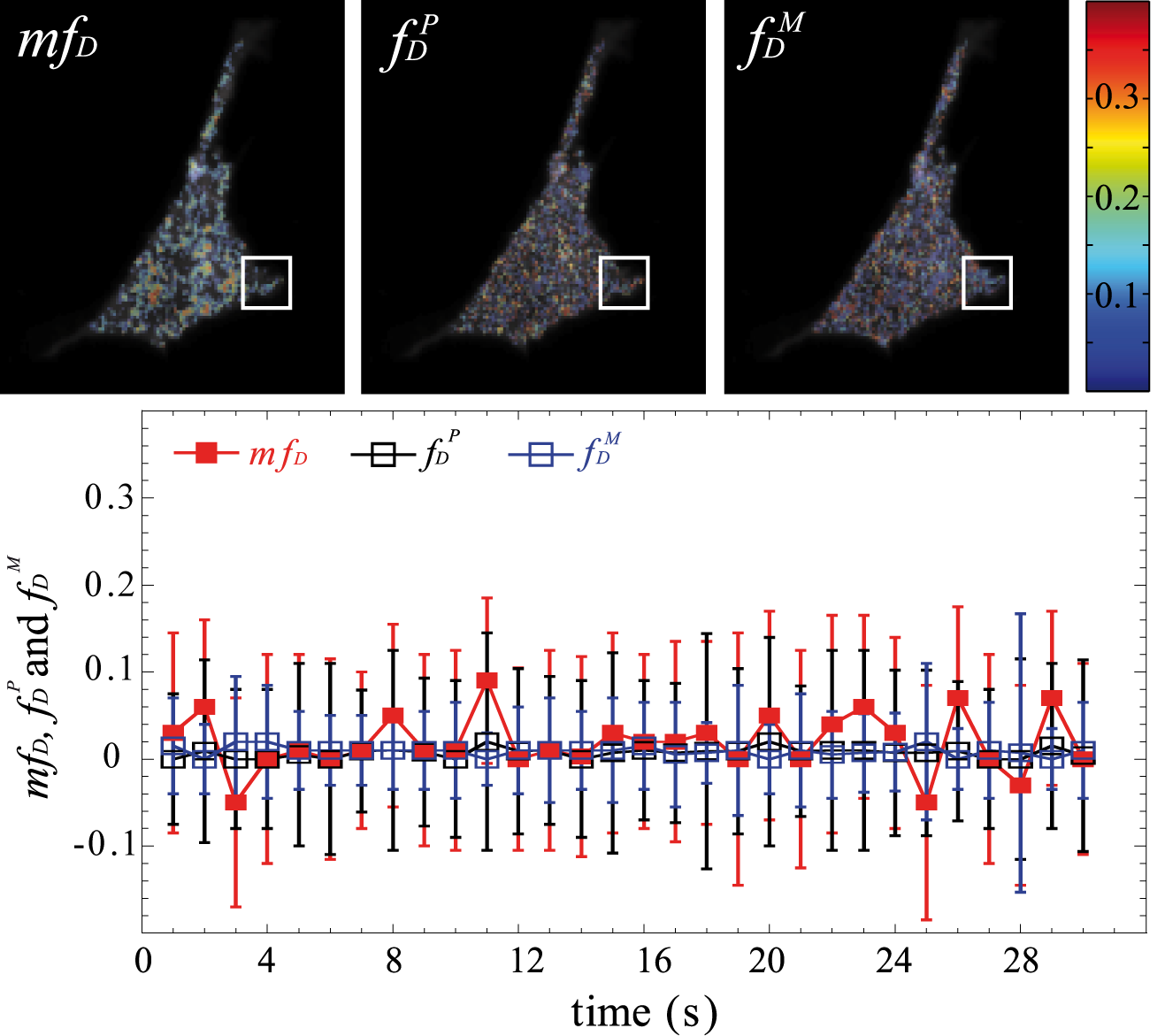

Supplement: Figure S5 — Calculations of mfD, fDP and fDM in a cell co-expressing Rac-GFP+mCherry (negative control). We have reported the corresponding images of mfD, fDP and fDM in the upper part of the figure. The evolution of each parameter calculated in the white region of interest is also plotted in the lower part. Markers with error bars represent the medians and interquartile ranges of each parameter. (TIF) [file pone.0069335.s005.tif]
