# Supplementary material for: 827Spatio-Temporal Quantification of FRET in Living Cells by Fast Time-Domain FLIM: A Comparative Study of Non-Fitting Methods
Source: PLoS One. 2013 Jul 18;8(7):e69335. doi: 10.1371/journal.pone.0069335 (PMC3715500; doi:10.1371/journal.pone.0069335)
Supplement: Text S1 — Theoretical calculations of the means and the standard deviations as a function of the number of photons. (DOC) [file pone.0069335.s006.doc]

**Text S1: Theoretical calculations of the means and the standard deviations as a function of the number of photons**

For estimating the expectation of *mf_D_*, as explained in the exhaustive work performed by Philip & Carlson , we write

$\tau=\frac{X}{Y}=\frac{\mu_{X}+\sigma_{X}Y_{X}}{\mu_{Y}+\sigma_{Y}Y_{Y}}=\frac{\mu_{X}\left(1+\kappa_{X}Y_{X}\right)}{\mu_{Y}\left(1+\kappa_{Y}Y_{Y}\right)}$ (A1)

In this expression, *µ_X_* and *µ_Y_* are the means and *σ_X_* and *σ_Y_* are the standard deviations of *X* and *Y* respectively; the notations *κ_X_*=*σ_X_/µ_X_* and *κ_Y_*=*σ_Y_/µ_Y_* are introduced and *Y_X_* and *Y_Y_* are random variables with mean zero and standard deviation 1. For calculating the expectation of *mf_D_,* we insert Eq. A1 into Eq. 3 and use a series expansion assuming that *κ_X_* and *κ_Y_* are (much) smaller than unity. By using: *E{Y_X_}*=0, *E{Y_Y_}*=0 and *E{Y_X_^2^}*=1, *E{Y_Y_^2^}*=1 and ignoring terms of order greater than 2, we obtain the following expectation for *mf_D_*

$E\left\{mf_{D}\right\}=1-\frac{\mu_{X}^{2}}{\left(\mu_{X}-2\mu_{Y}\tau_{D}^{2}\right)^{2}}-\frac{4\mu_{Y}\mu_{X}^{2}\tau_{D}\left(\mu_{X}+\mu_{Y}\tau_{D}\right)\kappa_{X}^{2}}{\left(\mu_{X}-2\mu_{Y}\tau_{D}^{2}\right)^{4}}+\frac{4\mu_{Y}\mu_{X}^{2}\tau_{D}\left(\mu_{X}+4\mu_{Y}\tau_{D}\right)\rho_{XY}\kappa_{X}\kappa_{Y}}{\left(\mu_{X}-2\mu_{Y}\tau_{D}^{2}\right)^{4}}-\frac{12\mu_{X}^{2}\mu_{Y}^{2}\tau_{D}^{2}\kappa_{Y}^{2}}{\left(\mu_{X}-2\mu_{Y}\tau_{D}^{2}\right)^{4}}$ (A2)

For a biexponential intensity decay with an infinite measurement window, the means, the standard deviations and the expectation of the crossed term are given by

$\mu_{X}=E\left\{X\right\}=N\int_{0}^{\infty} p(t)\times tdt=N\left(\phi_{D}\tau_{F}+(1-\phi_{D})\tau_{D}\right)$ (A3)

$\mu_{Y}=E\left\{Y\right\}=N\int_{0}^{\infty} p(t)dt=N$ (A4)

$\sigma_{x}^{2}=E\left\{X^{2}\right\}=N\int_{0}^{\infty} p(t)\times t^{2}dt=N\left(2\phi_{D}\tau_{F}^{2}+2(1-\phi_{D})\tau_{D}^{2}\right)$ (A5)

$\sigma_{Y}^{2}=E\left\{Y^{2}\right\}=N\int_{0}^{\infty} p(t)dt=N$ (A6)

$\rho_{XY}\sigma_{X}\sigma_{Y}=E\left\{XY\right\}=N\int_{0}^{\infty} p(t)\times tdt=N\left(\phi_{D}\tau_{F}+(1-\phi_{D})\tau_{D}\right)$ (A7)

Inserting Eqs. A3-A7 into Eq. A2 leads to

$E\left\{mf_{D}\right\}=\frac{4\phi_{D}\tau_{D}\left(\tau_{D}-\tau_{F}\right)}{\left(\tau_{D}+\phi_{D}\left(\tau_{D}-\tau_{F}\right)\right)^{2}}\left(1+\frac{\phi_{D}\tau_{D}\left(\tau_{D}^{2}+3\tau_{F}\tau_{D}-4\tau_{F}^{2}\right)-2\tau_{D}^{3}+\left(\phi_{D}^{3}-2\phi_{D}^{2}\right)\left(\tau_{F}-\tau_{D}\right)^{3}}{N\phi_{D}\left(\tau_{D}-\tau_{F}\right)\left(\tau_{D}+\phi_{D}\left(\tau_{D}-\tau_{F}\right)\right)^{2}}\right)$ (A8)

We use the same procedure for calculating the expectation of *mf_D_^2^* and we can finally calculate the standard deviation of *mf_D_* which is defined by

$\sigma\left\{mf_{D}\right\}=\sqrt{E\left\{mf_{D}^{2}\right\}-E\left\{mf_{D}\right\}^{2}}$ (A9)

After simplification, a straightforward calculation leads to

$\sigma\left\{mf_{D}\right\}=\frac{4\tau_{D}\left(\phi_{D}\tau_{F}+\left(1-\phi_{D}\right)\tau_{D}\right)\sqrt{\begin{matrix} \tau_{D}^{2}-\phi_{D}^{2}\left(\tau_{F}-\tau_{D}\right)^{2} \\ +2\phi_{D}\tau_{F}\left(\tau_{F}-\tau_{D}\right) \end{matrix}}}{\sqrt{N}\left(\tau_{D}+\phi_{D}\left(\tau_{D}-\tau_{F}\right)\right)^{3}}\sqrt{1+\frac{\begin{matrix} \left(\phi_{D}\left(\tau_{F}-\tau_{D}\right)+2\tau_{D}^{2}\right)^{2}\times\\ \left(\phi_{D}^{2}\left(\tau_{F}-\tau_{D}\right)^{2}-\tau_{D}^{2}+2\phi_{D}\tau_{F}\left(\tau_{D}-\tau_{F}\right)\right) \end{matrix}}{N\left(\tau_{D}^{2}-\phi_{D}^{2}\left(\tau_{F}-\tau_{D}\right)^{2}\right)^{2}}}$ (A10)

**Reference**

1. Philip J, Carlsson K (2003) Theoretical investigation of the signal-to-noise ratio in fluorescence lifetime imaging. J Opt Soc Am A 20: 368-379.
