# Supplementary material for: 827Spatio-Temporal Quantification of FRET in Living Cells by Fast Time-Domain FLIM: A Comparative Study of Non-Fitting Methods
Source: PLoS One. 2013 Jul 18;8(7):e69335. doi: 10.1371/journal.pone.0069335 (PMC3715500; doi:10.1371/journal.pone.0069335)
Supplement: Text S2 — Corrected expressions of the non fitting approaches. (DOC) [file pone.0069335.s007.doc]

**Text S2: Corrected expressions of the non fitting approaches**

The polar representation consists in calculating the [*u*; *v*] coordinates which are defined as the integrals of the product between the fluorescence intensity decay and the sine and cosine functions (Eqs. 5-6). In practice, these integrals are numerically approximated because the intensity decay is composed of a finite number of experimental points. This topic has already been exhaustively detailed for the polar approach by us . Briefly, by approximating these integrals with a sum of trapezoids, the corrected polar coordinates for an ideal mono-exponential intensity decay can be expressed as

$u=\left(1+\omega^{2}\tau_{m}^{2}\right)^{-1/2}\cos\left(\arctan\left(\omega\tau_{\phi}\right)\right)$ (B1)

$v=\left(1+\omega^{2}\tau_{m}^{2}\right)^{-1/2}\sin\left(\arctan\left(\omega\tau_{\phi}\right)\right)$ (B2)

where *τ_φ_* and *τ_m_* are the corrected phase and modulation lifetimes given by

$\tau_{\phi}=\frac{dt}{\text{arcsinh}\left(\sin\left(2\pi/N_{ch}\right)/\tan\phi^{\exp}\right)}$ (B3)

(B4)

where *φ_exp_* and *m_exp_* are respectively the phase and modulation measured experimentally and *dt* is the temporal width of the channels.

We have applied the same framework for determining the corrected mean lifetime. With trapezoidal approximation, the mean lifetime (for a mono-exponential intensity decay) is given by

$\left\langle\tau\right\rangle=\left[\frac{N_{ch}dt}{2}\exp\left(-\frac{N_{ch}dt}{\tau}\right)+\sum_{t=1}^{N_{ch}-1} \exp\left(-\frac{tdt}{\tau}\right)\times tdt\right]/\left[\frac{1}{2}\left(1+\exp\left(-\frac{N_{ch}dt}{\tau}\right)\right)+\sum_{t=1}^{N_{ch}-1} \exp\left(-\frac{tdt}{\tau}\right)\right]$ (B5)

which, from the exponential sum formulas, leads to

$\left\langle\tau\right\rangle=\frac{dt\left(N_{ch}-2\exp\left(dt/\tau\right)+2\exp\left(dt\left(N_{ch}+1\right)/\tau\right)-N_{ch}\exp\left(2dt/\tau\right)\right)}{\left(1-\exp\left(2dt/\tau\right)\right)\left(1-\exp\left(N_{ch}dt/\tau\right)\right)}$ (B6)

For calculating the corrected mean lifetime, we need to resolve the equality <*τ>*=*τ^exp^* where *τ^exp^* is the experimental measurement of the mean lifetime. However this equality is not resolvable analytically. We have then approximated the exponential functions with Maclaurin series and ignored terms of order greater than 10. It becomes now possible to resolve the equality <*τ>*=*τ^exp^*. We found that the corrected mean lifetime is the only real positive root of a polynomial of order 18 whose coefficients are reported in the supplementary Text S3.

We have finally used the same procedure for calculating the corrected moment of second order. With trapezoidal approximation, the second moment is defined for a mono-exponential intensity decay by

$E\left\{\tau^{2}\right\}=\left[\frac{N_{ch}^{2}dt^{2}}{2}\exp\left(-\frac{N_{ch}dt}{\tau}\right)+\sum_{t=1}^{N_{ch}-1} \exp\left(-\frac{tdt}{\tau}\right)\times t^{2}dt^{2}\right]/\left[\frac{1}{2}\left(1+\exp\left(-\frac{N_{ch}dt}{\tau}\right)\right)+\sum_{t=1}^{N_{ch}-1} \exp\left(-\frac{tdt}{\tau}\right)\right]$ (B7)

From the exponential sum formulas, these expressions can be written as

$E\left\{\tau^{2}\right\}=\frac{dt^{2}\left(\begin{matrix} N_{ch}^{2}+\left(2-N_{ch}\left(4+N_{ch}\right)\right)\exp\left(dt/\tau\right)+\left(2-N_{ch}\left(N_{ch}-4\right)\right)\exp\left(2dt/\tau\right) \\ +N_{ch}^{2}\exp\left(3dt/\tau\right)-2\exp\left(dt\left(N_{ch}+1\right)/\tau\right)-2\exp\left(dt\left(N_{ch}+2\right)/\tau\right) \end{matrix}\right)}{\left(\exp\left(dt/\tau\right)-1\right)^{2}\left(1+\exp\left(dt/\tau\right)\right)\left(1-\exp\left(N_{ch}dt/\tau\right)\right)}$ (B8)

We have then deduced the corrected second moment by approximating the exponential functions with Maclaurin series and ignored terms of order greater than 10 and by resolving the equality *E{τ^2^}*=*σ^exp^* where *σ^exp^* is the experimental measurement of the second moment. The corrected moment of second order is equal to the only real positive root of a polynomial of order 17 whose coefficients are indicated in the supplementary Text S4. These corrected expressions of *E{τ}=*<*τ>* and *E{τ^2^}* are used for estimating with the moments method the fraction of interacting donor *f_D_^M^* and the donor lifetime in presence of the acceptor *τ_F_^M^* (Eqs. 11 and 12).

Note that there is an additional correction for the expressions of the first and second order moments. Due to the finite width of the measurement window *T*, the mean lifetime <*τ*> and the second moment *E{τ^2^}* are given by

$\left\langle\tau\right\rangle=E\left\{\tau\right\}=\int_{0}^{T} t\times\exp\left(-\frac{t}{\tau}\right)dt/\int_{0}^{T} \exp\left(-\frac{t}{\tau}\right)dt=\tau-\frac{T}{\exp\left(\frac{T}{\tau}\right)-1}$ (B9)

$E\left\{\tau^{2}\right\}=\int_{0}^{T} t^{2}\times\exp\left(-\frac{t}{\tau}\right)dt/\int_{0}^{T} \exp\left(-\frac{t}{\tau}\right)dt=2\tau^{2}-\frac{T\left(T+2\tau\right)}{\exp\left(\frac{T}{\tau}\right)-1}$ (B10)

In order to take into account these additional terms, we have used the same protocols; we have replaced the exponential functions with Maclaurin series, omitted terms of order greater than 10 and resolved the equalities: <*τ>*=*τ^exp^* and *E{τ^2^}*=2(*τ^exp^*)*^2^*. The final <*τ*> and *E{τ^2^}* are the only real positive root of a polynomial equation of order 9.

**Reference**

1. Leray A, Spriet C, Trinel D, Usson Y, Héliot L (2012) Generalization of the Polar Representation in Time Domain Fluorescence Lifetime Imaging Microscopy for Biological Applications: practical implementation. J Microsc.
