# Supplementary material for: 827Spatio-Temporal Quantification of FRET in Living Cells by Fast Time-Domain FLIM: A Comparative Study of Non-Fitting Methods
Source: PLoS One. 2013 Jul 18;8(7):e69335. doi: 10.1371/journal.pone.0069335 (PMC3715500; doi:10.1371/journal.pone.0069335)
Supplement: Text S3 — Corrected expression of the mean lifetime. (DOC) [file pone.0069335.s008.doc]

**Text S3: Corrected expression of the mean lifetime**

For resolving the equality <*τ>*=*τ^exp^* (cf. Eq. B6), we have replaced the exponential functions with Maclaurin series and omitted terms of order greater than 10. We obtain that the corrected mean lifetime is the only real positive root of a polynomial equation *p* of order 18 which is defined by

$p=\sum_{i=0}^{18} a_{i}x^{i}$ (C1)

where *a_i_* are the coefficients which are equal to

$a_{0}=2\tau^{\exp}N_{ch}^{9}dt^{18}$ (C2)

$a_{1}=\tau^{\exp}N_{ch}^{8}dt^{17}\left(20+10N_{ch}\right)$ (C3)

$a_{2}=\tau^{\exp}N_{ch}^{7}dt^{16}\left(180+100N_{ch}+45N_{ch}^{2}\right)$ (C4)

$a_{3}=\tau^{\exp}N_{ch}^{6}dt^{15}\left(1440+900N_{ch}+450N_{ch}^{2}+180N_{ch}^{3}\right)$ (C5)

$a_{4}=\tau^{\exp}N_{ch}^{5}dt^{14}\left(10080+7200N_{ch}+4050N_{ch}^{2}+1800N_{ch}^{3}+630N_{ch}^{4}\right)$ (C6)

$a_{5}=\tau^{\exp}N_{ch}^{4}dt^{13}\left(60480+50400N_{ch}+32400N_{ch}^{2}+16200N_{ch}^{3}+6300N_{ch}^{4}+1890N_{ch}^{5}\right)$ (C7)

$a_{6}=\tau^{\exp}N_{ch}^{3}dt^{12}\left(\begin{matrix} 302400+302400N_{ch}+226800N_{ch}^{2}+129600N_{ch}^{3}+56700N_{ch}^{4}+18900N_{ch}^{5} \\ +4725N_{ch}^{6} \end{matrix}\right)$ (C8)

$a_{7}=\tau^{\exp}N_{ch}^{2}dt^{11}\left(\begin{matrix} 1209600+1512000N_{ch}+1360800N_{ch}^{2}+907200N_{ch}^{3}+453600N_{ch}^{4} \\ +170100N_{ch}^{5}+47250N_{ch}^{6}+9450N_{ch}^{7} \end{matrix}\right)$ (C9)

$a_{8}=\tau^{\exp}N_{ch}dt^{10}\left(\begin{matrix} 3628800+6048000N_{ch}+6804000N_{ch}^{2}+5443200N_{ch}^{3}+3175200N_{ch}^{4} \\ +1360800N_{ch}^{5}+425250N_{ch}^{6}+94500N_{ch}^{7}+14175N_{ch}^{8} \end{matrix}\right)$ (C10)

$a_{9}=\tau^{\exp}dt^{9}\left(\begin{matrix} 7257600+18144000N_{ch}+27216000N_{ch}^{2}+27216000N_{ch}^{3}+19051200N_{ch}^{4} \\ +9525600N_{ch}^{5}+3402000N_{ch}^{6}+850500N_{ch}^{7}+141750N_{ch}^{8}+14175N_{ch}^{9} \end{matrix}\right)$ (C11)

$a_{10}=dt^{8}\left(\begin{matrix} 36288000\tau^{\exp}+7115850dt+81648000\tau^{\exp}N_{ch}-637875dtN_{ch}+108864000\tau^{\exp}N_{ch}^{2} \\ -1701000dtN_{ch}^{2}+95256000\tau^{\exp}N_{ch}^{3}-2976750dtN_{ch}^{3}+57153600\tau^{\exp}N_{ch}^{4} \\ -3572100dtN_{ch}^{4}+23814000\tau^{\exp}N_{ch}^{5}-2976750dtN_{ch}^{5}+6804000\tau^{\exp}N_{ch}^{6}-1701000dtN_{ch}^{6} \\ +1275750\tau^{\exp}N_{ch}^{7}-637875dtN_{ch}^{7}+141750\tau^{\exp}N_{ch}^{8}-141750dtN_{ch}^{8}-14175dtN_{ch}^{9} \end{matrix}\right)$ (C12)

$a_{11}=dt^{7}\left(\begin{matrix} 163296000\tau^{\exp}+35012250dt+326592000\tau^{\exp}N_{ch}-5103000dtN_{ch}+381024000\tau^{\exp}N_{ch}^{2} \\ -11907000dtN_{ch}^{2}+285768000\tau^{\exp}N_{ch}^{3}-17860500dtN_{ch}^{3}+142884000\tau^{\exp}N_{ch}^{4} \\ -17860500dtN_{ch}^{4}+47628000\tau^{\exp}N_{ch}^{5}-11907000dtN_{ch}^{5}+10206000\tau^{\exp}N_{ch}^{6} \\ -5103000dtN_{ch}^{6}+1275750\tau^{\exp}N_{ch}^{7}-1275750dtN_{ch}^{7}-141750dtN_{ch}^{8} \end{matrix}\right)$ (C13)

$a_{12}=dt^{6}\left(\begin{matrix} 653184000\tau^{\exp}+153090000dt+1143072000\tau^{\exp}N_{ch}-35721000dtN_{ch} \\ +1143072000\tau^{\exp}N_{ch}^{2}-71442000dtN_{ch}^{2}+714420000\tau^{\exp}N_{ch}^{3}-89302500dtN_{ch}^{3} \\ +285768000\tau^{\exp}N_{ch}^{4}-71442000dtN_{ch}^{4}+71442000\tau^{\exp}N_{ch}^{5}-35721000dtN_{ch}^{5} \\ +10206000\tau^{\exp}N_{ch}^{6}-10206000dtN_{ch}^{6}-1275750dtN_{ch}^{7} \end{matrix}\right)$ (C14)

$a_{13}=dt^{5}\left(\begin{matrix} 2286144000\tau^{\exp}+581742000dt+3429216000\tau^{\exp}N_{ch}-214326000dtN_{ch} \\ +2857680000\tau^{\exp}N_{ch}^{2}-357210000dtN_{ch}^{2}+1428840000\tau^{\exp}N_{ch}^{3}-357210000dtN_{ch}^{3} \\ +428652000\tau^{\exp}N_{ch}^{4}-214326000dtN_{ch}^{4}+71442000\tau^{\exp}N_{ch}^{5}-71442000dtN_{ch}^{5} \\ -10206000dtN_{ch}^{6} \end{matrix}\right)$ (C15)

$a_{14}=dt^{4}\left(\begin{matrix} 6858432000\tau^{\exp}+1857492000dt+8573040000\tau^{\exp}N_{ch}-1071630000dtN_{ch} \\ +5715360000\tau^{\exp}N_{ch}^{2}-1428840000dtN_{ch}^{2}+2143260000\tau^{\exp}N_{ch}^{3} \\ -1071630000dtN_{ch}^{3}+428652000\tau^{\exp}N_{ch}^{4}-428652000dtN_{ch}^{4}-71442000dtN_{ch}^{5} \end{matrix}\right)$ (C16)

$a_{15}=dt^{3}\left(\begin{matrix} 17146080000\tau^{\exp}+4715172000dt+17146080000\tau^{\exp}N_{ch}-4286520000dtN_{ch} \\ +8573040000\tau^{\exp}N_{ch}^{2}-4286520000dtN_{ch}^{2}+2143260000\tau^{\exp}N_{ch}^{3} \\ -2143260000dtN_{ch}^{3}-428652000dtN_{ch}^{4} \end{matrix}\right)$ (C17)

$a_{16}=dt^{2}\left(\begin{matrix} 34292160000\tau^{\exp}+8573040000dt+25719120000\tau^{\exp}N_{ch}-12859560000dtN_{ch} \\ +8573040000\tau^{\exp}N_{ch}^{2}-8573040000dtN_{ch}^{2}-2143260000dtN_{ch}^{3} \end{matrix}\right)$ (C18)

$a_{17}=dt\left(\begin{matrix} 51438240000\tau^{\exp}+8573040000dt+25719120000\tau^{\exp}N_{ch}-25719120000dtN_{ch} \\ -8573040000dtN_{ch}^{2} \end{matrix}\right)$ (C19)

$a_{18}=51438240000\tau^{\exp}-25719120000dtN_{ch}$ (C20)
