# Supplementary material for: 827Spatio-Temporal Quantification of FRET in Living Cells by Fast Time-Domain FLIM: A Comparative Study of Non-Fitting Methods
Source: PLoS One. 2013 Jul 18;8(7):e69335. doi: 10.1371/journal.pone.0069335 (PMC3715500; doi:10.1371/journal.pone.0069335)
Supplement: Text S4 — Corrected expression of the second moment. (DOC) [file pone.0069335.s009.doc]

**Text S4: Corrected expression of the second moment**

We have applied the same procedure for resolving the equality *E{τ^2^}*=*σ^exp^* (cf. Eq. B8). By replacing the exponential functions with Maclaurin series and omitting terms of order greater than 8, we found that the corrected second moment is the only real positive root of a polynomial equation *p* of order 17

$p=\sum_{i=0}^{17} a_{i}x^{i}$ (D1)

where *a_i_* are the coefficients defined by

$a_{0}=\sigma^{\exp}N_{ch}^{8}dt^{17}$ (D2)

$a_{1}=\sigma^{\exp}N_{ch}^{7}dt^{16}\left(8+12N_{ch}\right)$ (D3)

$a_{2}=\sigma^{\exp}N_{ch}^{6}dt^{15}\left(56+96N_{ch}+84N_{ch}^{2}\right)$ (D5)

$a_{3}=\sigma^{\exp}N_{ch}^{5}dt^{14}\left(336+672N_{ch}+672N_{ch}^{2}+424N_{ch}^{3}\right)$ (D6)

$a_{4}=\sigma^{\exp}N_{ch}^{4}dt^{13}\left(1680+4032N_{ch}+4704N_{ch}^{2}+3392N_{ch}^{3}+1632N_{ch}^{4}\right)$ (D7)

$a_{5}=\sigma^{\exp}N_{ch}^{3}dt^{12}\left(6720+20160N_{ch}+28224N_{ch}^{2}+23744N_{ch}^{3}+13056N_{ch}^{4}+4992N_{ch}^{5}\right)$ (D8)

$a_{6}=\sigma^{\exp}N_{ch}^{2}dt^{11}\left(\begin{matrix} 20160+80640N_{ch}+141120N_{ch}^{2}+142464N_{ch}^{3}+91392N_{ch}^{4} \\ +39936N_{ch}^{5}+12288N_{ch}^{6} \end{matrix}\right)$ (D9)

$a_{7}=\sigma^{\exp}N_{ch}dt^{10}\left(\begin{matrix} 40320+241920N_{ch}+564480N_{ch}^{2}+712320N_{ch}^{3}+548352N_{ch}^{4} \\ +279552N_{ch}^{5}+98304N_{ch}^{6}+24192N_{ch}^{7} \end{matrix}\right)$ (D10)

$a_{8}=\sigma^{\exp}N_{ch}dt^{9}\left(\begin{matrix} 483840+1693440N_{ch}+2849280N_{ch}^{2}+\text{ 2741760}N_{ch}^{3}+\text{ 1677312}N_{ch}^{4} \\ +\text{ 688128}N_{ch}^{5}+\text{ 193536}N_{ch}^{6}+\text{ 36864}N_{ch}^{7} \end{matrix}\right)$ (D11)

$a_{9}=\sigma^{\exp}N_{ch}dt^{8}\left(\begin{matrix} \text{ 3386880}+\text{ 8547840}N_{ch}+\text{ 10967040}N_{ch}^{2}+\text{ 8386560}N_{ch}^{3} \\ +\text{ 4128768}N_{ch}^{4}+\text{ 1354752}N_{ch}^{5}+\text{ 294912}N_{ch}^{6}+\text{ 41472}N_{ch}^{7} \end{matrix}\right)$ (D12)

$a_{10}=\sigma^{\exp}N_{ch}dt^{7}\left(\begin{matrix} \text{ 17095680}+\text{ 32901120}N_{ch}+\text{ 33546240}N_{ch}^{2}+\text{ 20643840}N_{ch}^{3} \\ +\text{ 8128512}N_{ch}^{4}+\text{ 2064384}N_{ch}^{5}+\text{ 331776}N_{ch}^{6}+\text{ 27648}N_{ch}^{7} \end{matrix}\right)$ (D13)

$a_{11}=\sigma^{\exp}N_{ch}dt^{6}\left(\begin{matrix} \text{ 65802240}+\text{ 100638720}N_{ch}+\text{ 82575360}N_{ch}^{2}+\text{ 40642560}N_{ch}^{3} \\ +\text{ 12386304}N_{ch}^{4}+\text{ 2322432}N_{ch}^{5}+\text{ 221184}N_{ch}^{6} \end{matrix}\right)$ (D14)

$a_{12}=dt^{5}\left(\begin{matrix} \text{ }-7105536 dt^{2}+201277440 \sigma^{\exp} N_{ch}-28532736 dt^{2} N_{ch}+247726080 \sigma^{\exp} N_{ch}^{2} \\ +40379904 dt^{2} N_{ch}^{2}+162570240 \sigma^{\exp} N_{ch}^{3}-51093504 dt^{2} N_{ch}^{3}+61931520 \sigma^{\exp} N_{ch}^{4} \\ -32901120 dt^{2} N_{ch}^{4}+13934592 \sigma^{\exp} N_{ch}^{5}-13934592 dt^{2} N_{ch}^{5}+1548288 \sigma^{\exp} N_{ch}^{6} \\ -3870720 dt^{2} N_{ch}^{6}-663552 dt^{2} N_{ch}^{7}-55296 dt^{2} N_{ch}^{8} \end{matrix}\right)$ (D15)

$a_{13}=dt^{4}\left(\begin{matrix} \text{ }-28532736 dt^{2}+495452160 \sigma^{\exp} N_{ch}-100638720 dt^{2} N_{ch}+487710720 \sigma^{\exp} N_{ch}^{2} \\ +88584192 dt^{2} N_{ch}^{2}+247726080 \sigma^{\exp} N_{ch}^{3}-131604480 dt^{2} N_{ch}^{3}+69672960 \sigma^{\exp} N_{ch}^{4} \\ -69672960 dt^{2} N_{ch}^{4}+9289728 \sigma^{\exp} N_{ch}^{5}-23224320 dt^{2} N_{ch}^{5}-4644864 dt^{2} N_{ch}^{6} \\ -442368 dt^{2} N_{ch}^{7} \end{matrix}\right)$ (D16)

$a_{14}=dt^{3}\left(\begin{matrix} -1548288 dt^{2}+975421440 \sigma^{\exp} N_{ch}-108380160 dt^{2} N_{ch}+743178240 \sigma^{\exp} N_{ch}^{2} \\ +119992320 dt^{2} N_{ch}^{2}+278691840 \sigma^{\exp} N_{ch}^{3}-278691840 dt^{2} N_{ch}^{3}+46448640 \sigma^{\exp} N_{ch}^{4} \\ -116121600 dt^{2} N_{ch}^{4}-27869184 dt^{2} N_{ch}^{5}-3096576 dt^{2} N_{ch}^{6} \end{matrix}\right)$ (D17)

$a_{15}=dt^{2}\left(\begin{matrix} -9289728 dt^{2}+1486356480 \sigma^{\exp} N_{ch}-195084288 dt^{2} N_{ch}+836075520 \sigma^{\exp} N_{ch}^{2} \\ +143990784 dt^{2} N_{ch}^{2}+185794560 \sigma^{\exp} N_{ch}^{3}-464486400 dt^{2} N_{ch}^{3} \\ -139345920 dt^{2} N_{ch}^{4}-18579456 dt^{2} N_{ch}^{5} \end{matrix}\right)$ (D18)

$a_{16}=N_{ch}dt\left(\begin{matrix} \text{ }1672151040 \sigma^{\exp}-278691840 dt^{2}+557383680 \sigma^{\exp} N_{ch}+92897280 dt^{2} N_{ch} \\ -557383680 dt^{2} N_{ch}^{2}-92897280 dt^{2} N_{ch}^{3} \end{matrix}\right)$ (D19)

$a_{17}=N_{ch}\left( 1114767360 \sigma^{\exp}-185794560 dt^{2}-371589120 dt^{2} N_{ch}^{2}\right)$ (D20)
